# The Diploid Genome Sequence of an Individual Human

**DOI:** 10.1371/journal.pbio.0050254

**Published:** 2007-09-04

**Authors:** Samuel Levy, Granger Sutton, Pauline C Ng, Lars Feuk, Aaron L Halpern, Brian P Walenz, Nelson Axelrod, Jiaqi Huang, Ewen F Kirkness, Gennady Denisov, Yuan Lin, Jeffrey R MacDonald, Andy Wing Chun Pang, Mary Shago, Timothy B Stockwell, Alexia Tsiamouri, Vineet Bafna, Vikas Bansal, Saul A Kravitz, Dana A Busam, Karen Y Beeson, Tina C McIntosh, Karin A Remington, Josep F Abril, John Gill, Jon Borman, Yu-Hui Rogers, Marvin E Frazier, Stephen W Scherer, Robert L Strausberg, J. Craig Venter

**Affiliations:** 1 J. Craig Venter Institute, Rockville, Maryland, United States of America; 2 Program in Genetics and Genomic Biology, The Hospital for Sick Children, and Molecular and Medical Genetics, University of Toronto, Toronto, Ontario, Canada; 3 Department of Computer Science and Engineering, University of California San Diego, La Jolla, California, United States of America; 4 Genetics Department, Facultat de Biologia, Universitat de Barcelona, Barcelona, Catalonia, Spain; Lawrence Berkeley National Laboratories, United States of America

## Abstract

Presented here is a genome sequence of an individual human. It was produced from ∼32 million random DNA fragments, sequenced by Sanger dideoxy technology and assembled into 4,528 scaffolds, comprising 2,810 million bases (Mb) of contiguous sequence with approximately 7.5-fold coverage for any given region. We developed a modified version of the Celera assembler to facilitate the identification and comparison of alternate alleles within this individual diploid genome. Comparison of this genome and the National Center for Biotechnology Information human reference assembly revealed more than 4.1 million DNA variants, encompassing 12.3 Mb. These variants (of which 1,288,319 were novel) included 3,213,401 single nucleotide polymorphisms (SNPs), 53,823 block substitutions (2–206 bp), 292,102 heterozygous insertion/deletion events (indels)(1–571 bp), 559,473 homozygous indels (1–82,711 bp), 90 inversions, as well as numerous segmental duplications and copy number variation regions. Non-SNP DNA variation accounts for 22% of all events identified in the donor, however they involve 74% of all variant bases. This suggests an important role for non-SNP genetic alterations in defining the diploid genome structure. Moreover, 44% of genes were heterozygous for one or more variants. Using a novel haplotype assembly strategy, we were able to span 1.5 Gb of genome sequence in segments >200 kb, providing further precision to the diploid nature of the genome. These data depict a definitive molecular portrait of a diploid human genome that provides a starting point for future genome comparisons and enables an era of individualized genomic information.

## Author Summary

We have generated an independently assembled diploid human genomic DNA sequence from both chromosomes of a single individual (J. Craig Venter). Our approach, based on whole-genome shotgun sequencing and using enhanced genome assembly strategies and software, generated an assembled genome over half of which is represented in large diploid segments (>200 kilobases), enabling study of the diploid genome. Comparison with previous reference human genome sequences, which were composites comprising multiple humans, revealed that the majority of genomic alterations are the well-studied class of variants based on single nucleotides (SNPs). However, the results also reveal that lesser-studied genomic variants, insertions and deletions, while comprising a minority (22%) of genomic variation events, actually account for almost 74% of variant nucleotides. Inclusion of insertion and deletion genetic variation into our estimates of interchromosomal difference reveals that only 99.5% similarity exists between the two chromosomal copies of an individual and that genetic variation between two individuals is as much as five times higher than previously estimated. The existence of a well-characterized diploid human genome sequence provides a starting point for future individual genome comparisons and enables the emerging era of individualized genomic information.

## Abstract 3

Comparison of the DNA sequence of an individual human from the reference sequence reveals a surprising amount of difference.

## Introduction

Each of our genomes is typically composed of DNA packaged into two sets of 23 chromosomes; one set inherited from each parent whose own DNA is a mosaic of preceding ancestors. As such, the human genome functions as a diploid entity with phenotypes arising due to the sometimes complex interplay of alleles of genes and/or their noncoding functional regulatory elements.

The diploid nature of the human genome was first observed as unbanded and banded chromosomes over 40 years ago [1–4] , and karyotyping still predominates in clinical laboratories as the standard for global genome interrogation. With the advent of molecular biology, other techniques such as chromosomal fluorescence in situ hybridization (FISH) and microarray-based genetic analysis [5,6] provided incremental increases in the resolution of genome analysis. Notwithstanding these approaches, we suspect that only a small proportion of genetic variation is captured for any sample in any one set of experiments.

Over the past decade, with the development of high-throughput DNA sequencing protocols and advanced computational analysis methods, it has been possible to generate assemblies of sequences encompassing the majority of the human genome [7–9]. Two versions of the human genome currently available are products of the Human Genome Sequencing Consortium [9] and Celera Genomics [7], derived from clone-based and random whole genome shotgun sequencing strategies, respectively. The Human Genome Sequencing Consortium assembly is a composite derived from haploids of numerous donors, whereas the Celera version of the genome is a consensus sequence derived from five individuals. Both versions almost exclusively report DNA variation in the form of single nucleotide polymorphisms (SNPs). However smaller-scale (<100 bp) insertion/deletion sequences (indels) or large-scale structural variants [10–15] also contribute to human biology and disease [16–18] and warrant an extensive survey.

The ongoing analyses of these DNA sequence resources have offered an unprecedented glimpse into the genetic contribution to human biology. The simplification of our collective genetic ancestry to a linear sequence of nucleotide bases has permitted the identification of functional sequences to be made primarily through sequence-based searching alignment tools. This revealed an unexpected paucity of protein coding genes (20,000–25,000) residing in less than 2% of the DNA examined, suggesting that alternative transcription and splicing of genes are equally important in development and differentiation [19,20]. The sequencing of DNA of various eukaryotic genomes, such as for murine [21,105] and primate [22,23] as well as many others, has enabled a comparative genomics strategy to refine the identification of orthologous genes. These genomic datasets have also enabled the identification of additional functional sequence such as *cis*-regulatory DNA [24–29] as well as both noncoding and microRNA [30–34] .

Building on the existing genome assemblies, numerous initiatives have explored variation at the population level, in particular to generate markers and maps as a means of understanding how sequence variation evolves and can contribute to phenotype. The initial drafts of the two human genomes provided an excess of 2.4 million SNPs [7,8] providing a platform for the initial phase of the HapMap project [35]. This ambitious project initially catalogued genetic variation at more than 1.2 million loci in 269 humans of four ethnicities, enabling a definition of common haplotypes and resulting in tag SNP sets for these populations. The use of these data has already allowed the mapping and identification of susceptibility genes and loci involved in complex diseases such as asthma [36], age related macular degeneration [37], and type II diabetes [38]. Notwithstanding, there are limitations with current SNP-based genome-wide association studies, because they rely on reconstructing haplotypes based on population data and can be uninformative or misleading in regions of low linkage disequilibrium (LD). Further, association studies have been designed to detect common disease variants and are not optimized to detect rare etiological variants [39].

The ability to generate a diploid genome structure via haplotype phasing for the HapMap samples is limited by the SNPs that were genotyped and their spacing. By using LD measures, it was possible to identify diploid blocks of DNA averaging 16.3 kb for Caucasians (CEU), 7.3 kb for Yorubans (YRI), and 13.2 kb for grouped Han Chinese and Japanese (CHB+JPT) [35]. However, LD varies across the genome, and regions of low LD, i.e., high recombination, cannot be represented by haplotype blocks. Furthermore, these diploid blocks are incomplete because there may be unknown variants between the SNP loci sampled. These results do not permit a comprehensive definition of the sequence present at each allele nor the information that produces the relevant allelic combinations, which are essential in identifying the differences of biological information encoded by the diploid state. The ability to perform, in a practical manner, whole-genome sequencing in large disease populations would enable the construction of haplotypes from individuals' genomes, thus phasing all variant types throughout the genome without assumptions about population history. Clearly, to enable the forthcoming field of individualized genomic medicine, it is important to represent and understand the entire diploid genetic component of humans, including all forms of genetic variation in nucleotide sequences, as well as epigenetic effects.

To understand fully the nature of genetic variation in development and disease, indeed the ideal experiment would be to generate complete diploid genome sequences from numerous controls and cases. Here we report our endeavor to fully sequence a diploid human genome. We used an experimental design based on very high quality Sanger-based whole-genome shotgun sequencing, allowing us to maximize coverage of the genome and to catalogue the vast majority of variation within it. We discovered some 4.1 million variants in this genome, 30% of which were not described previously, furthering our understanding of genetic individuality. These variants include SNPs, indels, inversions, segmental duplications, and more complex forms of DNA variation. We used the variant set coupled with the sequence read information and mate pairs to build long-range haplotypes, the boundaries of which provide coverage of 11,250 genes (58% of all genes). In this manner we achieved our goal of the construction of a diploid genome, which we hope will serve as a basis for future comparison as more individual genomes are produced.

## Results

### Donor Pedigree and Karyotype

The individual whose genome is described in this report is J. Craig Venter, who was born on 14 October 1946, a self-identified Caucasian male. The DNA donor gave full consent to provide his DNA for study via sequencing methods and to disclose publicly his genomic data in totality. The collection of DNA from blood with attendant personal, medical, and phenotypic trait data was performed on an ongoing basis. Ethical review of the study protocol was performed annually. Additionally, we provide here an initial foray into individualized genomics by correlating genotype with family history and phenotype; however, a more extensive analysis will be presented elsewhere.

**Figure 1:**
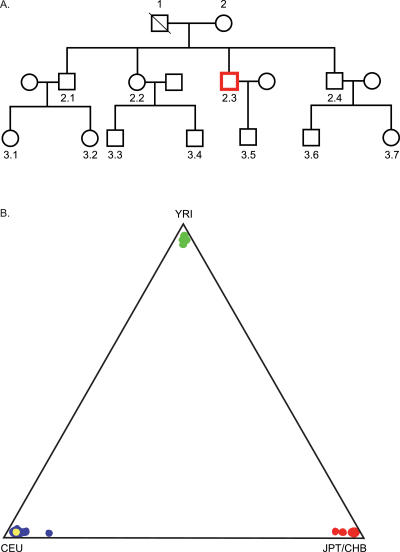
DNA Donor Pedigree and Relatedness to Ethnogeographic Populations (A) Three-generation pedigree showing the relation of ancestors to study DNA sample. The donor is identified in red. (B) Cluster analysis based on 750 SNP genotype information to infer the ancestry of the HuRef donor. The figure shows the proportion of membership of the HuRef donor (yellow) to three pre-defined HapMap populations (CEU = Northern and Western Europe, YRI = Yoruban, Ibadan, Nigeria, and JPT+CHB = Japanase, Tokyo, and Han Chinese, Beijing). The results indicate that the HuRef donor clusters with 99.5% similarity to the samples of northern and western European ancestry.

**Figure 2:**
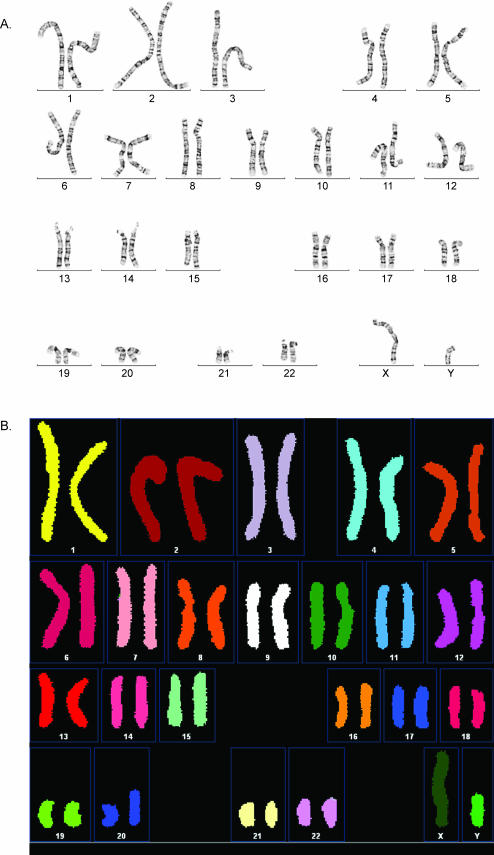
Results of Cytogenetic Analysis (A) HuRef donor G-banded karyotype. (B) Spectral karyotype analysis.

The donor's three-generation pedigree is shown in Figure 1A. The donor has three siblings and one biological son, his father died at age 59 of sudden cardiac arrest. There are documented cases of family members with chronic disease including hypertension and ovarian and skin cancer. According to the genealogical record, the donor's ancestors can be traced back to 1821 (paternal) and the 1700s (maternal) in England. Genotyping and cluster analysis of 750 unique SNP loci discovered through this project support that the donor is indeed 99.5% similar to individuals of European descent (Figure 1B), consistent with self-reporting. This is further corroborated by an extensive five-generation family history provided by the donor (unpublished data). Cytogenetic analysis through G-banded karyotyping and spectral karyotypic chromosome imaging reveals no obvious chromosomal abnormalities (Figure 2) that need to be considered in interpretation of genome assembly results or phenotypic association analyses.

### Genome Sequencing and Assembly

The assembly, herein referred to as HuRef, was derived of approximately 32 million sequence reads (Table S1) generated by a random shotgun sequencing approach using the open-source Celera Assembler. The approach used is similar in many respects to the whole-genome shotgun assembly (WGSA) reported previously [40], but there are three major differences: (i) HuRef was assembled entirely from shotgun reads from a single individual, whereas WGSA was based on shotgun reads from five individuals [7,40,41], albeit the majority of reads were from the same individual as HuRef; (ii) the approximate depth of sequence coverage for HuRef was 7.5 versus 5.3 for WGSA, although the clone coverage was about the same for both (Table 1) [7,40]; and (iii) the release of Celera Assembler as an open-source project has allowed us and others to continue to improve the assembly algorithms. As a consequence, we made modifications for the specification of consensus sequence differences found at distinct alleles. The multiple sequence alignment methodology was improved and reads were grouped by allele, thus allowing the determination of alternate consensus sequences at variant sites (see Materials and Methods).

**Table 1:**
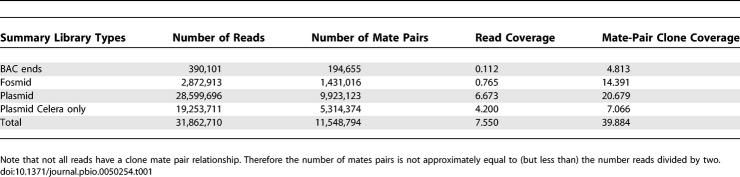
Clone Insert Library Types and Reads Used for HuRef Genome Assembly

HuRef is a high-quality draft genome sequence as evidenced from the contiguity statistics (Table 2). Improving the assembly algorithms and increasing the sequencing depth of coverage (compared to WGSA) resulted in a 68% decrease in the number of gaps within scaffolds from 206,552 (WGSA) to 66,815 (HuRef) as previously predicted [40]. We also observed a more than 4-fold increase in the N50 contig size (the length such that 50% of all base pairs are contained in contigs of the given length or larger) to 106 kb (HuRef) from 23 kb (WGSA). We used a fairly standard, but arbitrary, cutoff of 3,000 bp (similar to what was used for WGSA) to distinguish between scaffolds that were part of the HuRef assembly proper versus partially assembled and poorly incorporated sequence (see Materials and Methods). This resulted in 4,528 scaffolds (containing 2,810 Mb) of which 553 scaffolds were at least 100 kb in size (containing 2,780 Mb), whereas WGSA had 4,940 scaffolds (containing 2,696 Mb) of which 330 scaffolds were at least 100 kb (containing 2,669 Mb). The scaffold lengths for HuRef (N50 = 19.5 Mb) were somewhat shorter than WGSA (N50 = 29 Mb) primarily due to the difference in insert size for bacterial artificial chromosome (BAC) end mate pairs—HuRef 91 kb versus WGSA > 150 kb (Table 2) [41]. We determined that 144 of the 553 large HuRef scaffolds could be joined by two or more of the WGSA BAC mate pairs, and 98 more by a single WGSA BAC mate pair (see Materials and Methods), suggesting that use of large insert BAC libraries (>150 kb) would generate larger scaffolds.

**Table 2:**
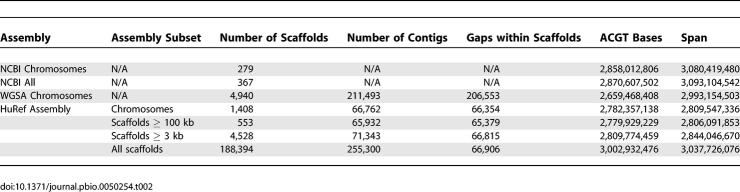
Summary of HuRef Assembly Statistics and Comparison to the Human NCBI Genome

### Assembly-to-Assembly Mapping

Genomic variation was observed by two approaches. First, we identified heterozygous alleles within the HuRef sequence. This variation represents differences in the maternal and paternal chromosomes. In addition, a comparison between HuRef and the National Center for Biotechnology Information (NCBI) version 36 human genome reference assembly, herein referred to as a one-to-one mapping, also served as a source for the identification of genomic variation. These comparisons identified a large number of putative SNPs as well as small, medium, and large insertion/deletion events and some major rearrangements described below. For the most part, the one-to-one mapping showed that both sequences are highly congruent with very large regions of contiguous alignment of high fidelity thus enabling the facile detection of DNA variation (Table S2).

The one-to-one mapping to NCBI version 36 (hereafter NCBI) was also used to organize HuRef scaffolds into chromosomes. HuRef scaffolds were only mapped to HuRef chromosomes if they had at least 3,000 bp that mapped and the scaffold was mostly not contained within a larger scaffold. With the exception of 12 chimeric joins, all scaffolds were placed in their entirety with no rearrangement onto HuRef chromosomes. The 12 chimeric regions represent the misjoining of a small number of chimeric scaffold/contigs by the Celera Assembly [40], as detected with mate pair patterns [7,42], and are also apparent by comparison to another assembly (Materials and Methods). The 12 chimeric joins in the HuRef scaffolds were split when these scaffolds were assigned to build HuRef chromosomes. Inversions and translocations within the nonchimeric scaffolds relative to NCBI are thus maintained within the HuRef chromosomes. The final set of 24 HuRef chromosomes were thus assembled from 1,408 HuRef assembly scaffolds and contain 2,782 Mb of ordered and oriented sequence.

The NCBI autosomes are on average 98.3% and 97.1% represented by runs and matches, respectively, in the one-to-one mapping to HuRef scaffolds (Table S3). A match is a maximal high-identity local alignment, usually terminated by indels or sequence gaps in one of the assemblies. Runs may include indels and are monotonically increasing or decreasing sets of matches (linear segments of a match dot plot) with no intervening matches from other runs on either axis.

**Figure 3:**
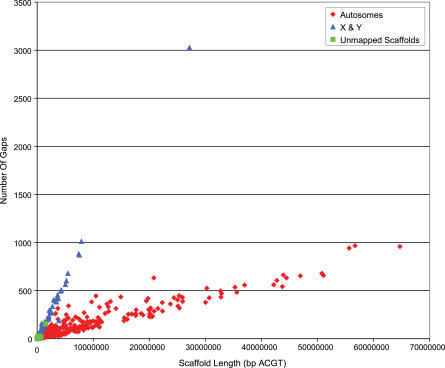
Sequencing Continuity Plot for the HuRef Autosomes Compared to HuRef X and Y Chromosomes Note that the autosomes have more contiguous sequence with fewer gaps compared to chromosomes X and Y, probably due to half the read depth compared to the autosomes and the presence of extensive sequence similarity between the sex chromosomes.

The Y chromosome is 59% covered by the one-to-one mapping due to difficulties when producing comparison between repeat rich chromosomes. In addition, the Y chromosome is more poorly covered because of the difficulties in assembling complex regions with sequencing depth of coverage only half that of the autosomal portion of the genome. The X chromosome coverage with HuRef scaffolds is at 95.2%, which is typical of the coverage level of autosomes (mean 98.3% using runs). However it is clear that the X chromosome has more gaps, as evidenced by the coverage with matches (89.4%) compared with the mean coverage of autosomes using matches (97.1%). The overall effects of lower sequence coverage on chromosomes X and Y are clearly evident as a sharp increase in number of gaps per unit length and shorter scaffolds compared to the autosomes (Figure 3). Similarity between the sex chromosomes is another source of assembly and mapping difficulties. For example, there is a 1.5-Mb scaffold that maps equally well to identical regions of the X and Y chromosomes and therefore cannot be uniquely mapped to either (see Materials and Methods and Figure 3). From our one-to-one mapping data, we are also able to detect the enrichment of large segmental duplications [10] on Chromosomes 9, 16, and 22, resulting in reduced coverage based on difficulties in assembly and mapping (Table S3).

Since NCBI, WGSA, and HuRef are all incomplete assemblies with sequence anomalies, assembly-to-assembly mappings also reflect issues of completeness and correctness. We compared three sets of chromosome sequences to evaluate this issue (see Materials and Methods): NCBI with the exclusion of the small amount of unplaced sequences, HuRef, and WGSA (Table S2) were thus compared in a pairwise manner. The comparison of WGSA and HuRef revealed 83 Mb more sequence in HuRef in matched segments of these genomes. This sequence is predominantly from HuRef that fills gaps in WGSA. Comparisons of HuRef and WGSA to NCBI showed the considerable improvement of HuRef over WGSA. Correspondingly, in HuRef there are approximately 120 Mb of additional aligned sequence, composed of 47 Mb of HuRef sequence that aligns to NCBI that was not aligned in WGSA and 73 Mb within aligned regions that fill gaps in WGSA. This comparison also showed an improvement factor of two in rearrangement differences (order and orientation) from WGSA to HuRef when mapped to the NCBI reference genome at small (<5 kb), medium (5–50 kb), and large (>50 kb) levels of resolution (Table S2). HuRef includes 9 Mb of unmatched sequence that fill gaps in NCBI or are identified as indel variants. An additional 14 Mb of HuRef chromosome sequence outside of aligned regions with NCBI represents previously unknown human genome sequence. The large regions of novel HuRef sequence are identified to be either: (a) gap filling or insertions, (b) unaligned NCBI chromosome regions, or (c) large scaffolds not mapped to NCBI chromosomes. Some of these were investigated using FISH analysis and are discussed below. Although we were able to organize HuRef scaffolds into HuRef chromosome sequence, all of the subsequent analyses in this report were accomplished using HuRef scaffold sequences.

### Identification of DNA Variants

#### Variant identification internal to the one-to-one map.

**Figure 4:**
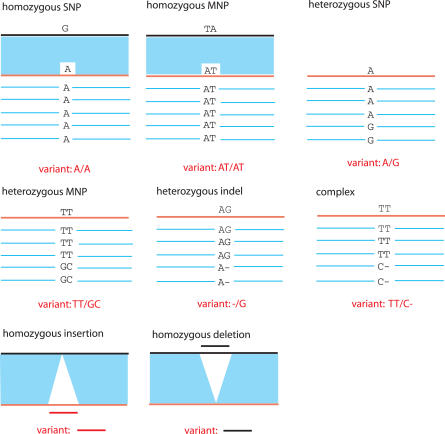
The Different Variant Types Identified from the HuRef Assembly and the HuRef-NCBI Assembly-to-Assembly Mapping HuRef consensus sequence (in red) with underlying sequence reads (in blue). Homozygous variants are identified by comparing the HuRef assembly with NCBI reference assembly. Heterozygous variants are identified by base differences between sequence reads. SNP = single nucleotide polymorphism; MNP = multi-nucleotide polymorphism, which contains contiguous mismatches.

The HuRef assembly and the one-to-one mapping between the HuRef genome and the NCBI reference genome resulted in the identification of 5,061,599 putative SNPs, heterozygous indels, and a variety of multi-nucleotide variations events (see Figure 4 for a definition), of which 62% are in the database for DNA variants (dbSNP; http://www.ncbi.nlm.nih.gov/SNP/). A significant fraction of these putative variants resulted from sequence reads with variant base having reduced quality value (QV) scores, the presence of variants in homopolymer runs and erroneous base calls at the beginning and end of reads. The inclusion of these reads was important to the assembly process, and therefore we chose to perform post-assembly processing to filter these variants to reduce false positives while limiting false negatives (column %red/%FN in Table 3 and detailed discussion in Material and Methods). The filters deemed most productive in creating a high-confidence variant set involved the application of a minimal QV threshold and testing for the location of a variant in sequence read. In addition, we applied the filter that a variant required supporting evidence from at least two reads and that the second allele had a minimum fraction of representative reads (20% reads with minor allele for heterozygous SNP and 25% for heterozygous indels). As indicated in Table 3, a significant improvement in reducing false positives while limiting false negatives is possible when the filters are applied independently on QV and read location–filtered variants. However, the maximum benefit from this filtering approach was achieved by applying filters cumulatively, and it was the three aforementioned filters (bold rows in Table 3) that were applied ultimately. After applying the filters, 81% of heterozygous indels, 29% of heterozygous SNPs, 7% of homozygous SNPs, and 19% homozygous indels were removed from the initial set. The filtering mainly affects heterozygous variants by reducing the number of reads that can be used for support. The cumulative application of the filters generated a set of variants from which a subset of 95,733 could be combined further into clusters. The first case where variants were clustered was when two SNPs were within 2 bp of each other. We clustered these, because there was more accuracy in classifying whether the variant caused a change in protein coding and not because they necessarily represent single mutational events. The second scenario for clustering involved non-SNP variants within 10 bp of other non-SNP variants, such as indels or complex variants. We decided to cluster these variants because extensive manual inspection showed that closely spaced indels were frequently better defined as one variant after realignment. Consequently, the clustering of variant positions was coupled with a localized realignment of sequence reads to define either two distinct alleles or haplotypes. Overall, the filtering and clustering refinements that were applied to the “raw” variant set resulted in a set of 3,325,530 variants within the one-to-one HuRef-to-NCBI mapping, of which 85% were found in dbSNP (Table 4).

**Table 3:**
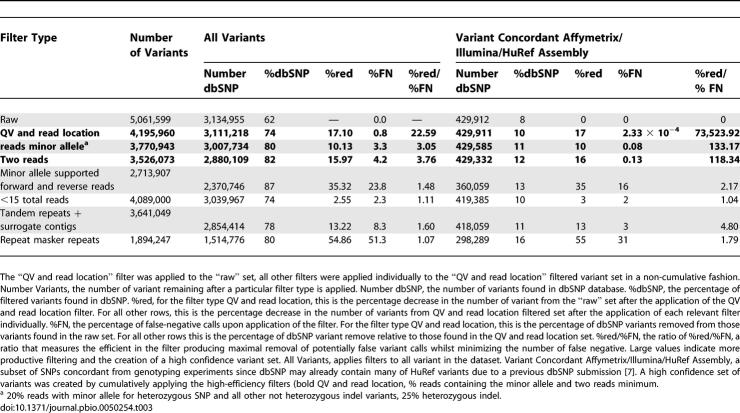
The Application of Distinct, Independent, Filtering Methods on the Detection Rate of SNPs, Heterozygous Indels, and Complex Variants Identified from the HuRef Assembly

**Table 4:**
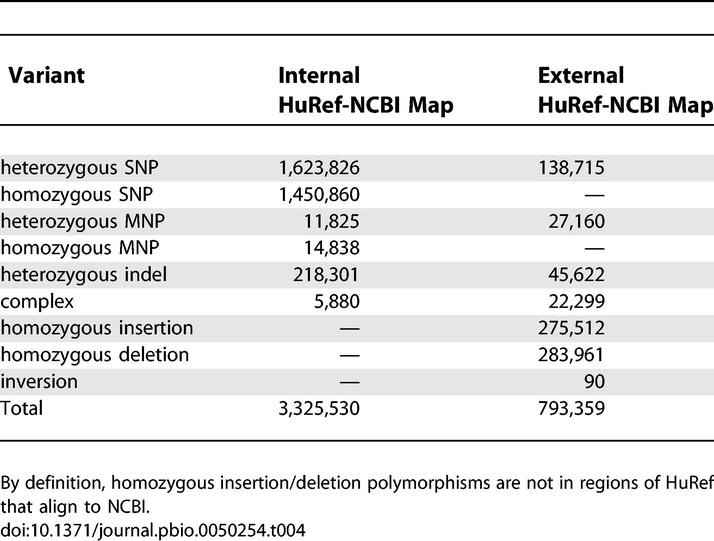
Identification of Variants Found within the HuRef-NCBI One-to-One Assembly Map (Internal HuRef-NCBI map) and Those Variants in HuRef Sequence Not Aligned to NCBI (External HuRef-NCBI Map)

#### Variant identification external to the one-to-one map.

The one-to-one mapping of HuRef to NCBI produced approximately 150 Mb of unaligned HuRef sequence inclusive of partially mapped and nonmapped HuRef scaffolds. Within this unaligned HuRef sequence, we identified 233,796 heterozygous variants including SNPs, indels, and complex variants after application of the same filters described above (see Table 4, variants labeled External HuRef-NCBI map). Other sources of variant external to the one-to-one mapping between the HuRef and NCBI human genome assemblies are putative homozygous insertions, deletions, and inversions (see Figure 4 for definitions), of which 693,941 were detected. This number of putative insertions and deletions was reduced by 19% by the application of a series of filters designed to eliminate the bulk of spurious variation. Therefore, variants were not called at the read margins (thresholds were the same as previously used for SNP and indels internal to the HuRef-NCBI map), and any identified variants required the supporting evidence of at least two reads and one satisfied mate pair with no ambiguous bases constituting the sequence of the insertion or deletion.

In addition to the aforementioned filtering approach, a small fraction (∼1%) of the 693,941 putative homozygous insertion/deletion variants were subsequently characterized as heterozygous variants. This was accomplished by finding exact matches of 100-bp sequence 5′ and 3′ of the insertion point sequence and the deletion sequence in both HuRef scaffolds and unassembled reads. This fraction of heterozygotes is likely to be a conservative estimate of the total number of true heterozygotes (see below). The alternate alleles of these heterozygous variants were primarily found (96% of the time) in scaffolds less than 5,000 bp long or in unassembled reads. This highlights the value of small scaffolds and unassembled reads in defining the variant set in an assembled genome and suggests that these elements are a rich source of genomic variation. Therefore, subsequent to the removal of the variants by read-based filtering (19% mentioned above) and the recategorization as heterozygous variants (1% above), the remaining variants included approximately equal numbers of insertion (275,512) and deletion (283,961) alleles and 90 inversions as outlined in Table 4.

In summary, using the combined identification and filtering approaches, it was possible to identify an initial “raw” set of 5,775,540 variants, from which we generated a higher-confidence set of 4,118,889 variants, of which 1,288,319 variants are novel relative to current databases (dbSNP).

### Initial Characterization of Variants

To examine sequence diversity in the genome, we estimated nucleotide diversity using the population mutation parameter θ [43]. This measure is corrected for sample size and the length of the region surveyed. In the case of a single genome with two chromosomes, θ simplifies to the number of heterozygote variants divided by the number of base pairs (see Materials and Methods). We define θ_SNP_ as the nucleotide diversity for SNPs (number of heterozygous SNPs/number of base pairs) and θ_indel_ as the diversity for indels (number of heterozygous indels/number of base pairs) [44]. For both θ_SNP_ and θ_indel_, the 95% confidence interval would be [0, 3θ] due to the small number of chromosomes (*n* = 2) being sampled (see Materials and Methods).

Across all autosomal chromosomes, the observed diversity values for SNPs and indels are 6.15 × 10^−4^ and 0.84 × 10^−4^ respectively. When restricted to coding regions only, θ_SNP_ = 3.59 × 10^−4^ and θ_indel_ = 0.07 × 10^−4^, indicating that 42% of SNPs and 91% of indels have been eliminated by selection in coding regions. The strong selection against coding indels is not surprising, because most will introduce a frameshift and produce a nonfunctional protein. Our observed θ_SNP_ falls within the range of 5.4 × 10^−4^ to 8.3 × 10^−4^ that has been previously reported by other groups [44–47].

Our observed θ_indel_ (0.84 × 10^−4^) is approximately 2-fold higher than the diversity value of 0.41 × 10^−4^ that was reported from SeattleSNPs (http://pga.gs.washington.edu), which was derived from directed resequencing of 330 genes in 23 individuals of European descent [44]. The values of θ_indel_ in repetitive sequence regions are 1.2 × 10^−4^ for regions identified by RepeatMasker (http://www.repeatmasker.org) and 4.9 × 10^−4^ for regions identified by TandemRepeatFinder [48], respectively. Thus, the indel diversity in repetitive regions is between 1.4 and 5.8 times higher than the genome-wide rate. This suggests that the high value of θ_indel_ over all loci is likely mediated by the abundance of indels in repetitive sequence. It is also possible that repetitive regions in genic sequence are under stronger selective pressure and therefore have lower indel diversity. These are precisely the regions that have been targeted in previous resequencing projects [44] from which indel diversity values have been determined. Additionally, repetitive regions also have more erroneous variant calls due to technical difficulties in sequencing and assembly of these types of regions. Therefore, our estimate for θ_indel_ is likely a combination of both a true higher mutation rate in repetitive regions and sequencing errors.

**Figure 5:**
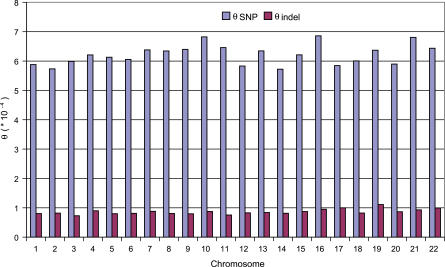
Diversity for SNPs and Indels in Autosomes This is most likely an under-estimate of the true diversity, because a fraction of real heterozygotes were missed due to insufficient read coverage.

Values of θ_indel_ are consistent among the chromosomes (Figure 5). Chromosomes with high θ_indel_ values also have a larger fraction of tandem repeats. For example, Chromosome 19 has the highest θ_indel_ (1.1 × 10^−4^ compared with the chromosomal average of 0.86 × 10^−4^), and it also has the highest proportion of tandem repeats (13% compared with the chromosomal average of 7%). The fraction of tandem repeats of a chromosome is positively correlated with the value of θ_indel_ for each chromosome (*r* = 0.73), so that the diversity of indels is associated with the underlying sequence composition.

The SNP variants identified in the HuRef genome include a larger-than-expected number of homozygous variants than those commonly observed in population-based studies (compare ratios of heterozygous SNP:homozygous SNP in Table 5). Our homozygous variants are detected as differences between the HuRef genome and the NCBI genome. One common interpretation of a homozygous variant is that given a common allele A and a rare allele B, the homozygous SNP is BB. However, because not all variant frequencies are known, we cannot determine if a position may carry the minor B allele in homozygous form. We analyzed ENCODE data using this definition and found the ratio of heterozygous SNPs to homozygous SNPs is 4.9 in an individual [49]. For our dataset, the observed ratio of heterozygous to homozygous SNP, where our “homozygous” SNPs are detected as bases differing from the NCBI human genome, is 1.2. To resolve this discrepancy, we examined the homozygous positions in the HuRef assembly and found that the increased frequency of homozygous SNPs results from the presence of minor alleles (BB) in the NCBI genome assembly. We observed that 75% of the homozygous positions in HuRef also had a SNP identified by the ENCODE [49]. A comparison of the alleles at these positions revealed that in 56% of the instances the HuRef genome had the more common allele, whereas the NCBI genome contained the minor allele. The remaining homozygous SNPs tended to be common minor alleles (76% had minor allele frequency [MAF] ≥ 0.30), consistent with their observation in homozygous form in the HuRef genome. Therefore, we confirmed that a large fraction of homozygous alleles from HuRef are real, and that differences between the HuRef and NCBI assemblies are due to NCBI containing the minor allele at a given SNP position, or HuRef containing a common SNP in homozygous form.

**Table 5:**
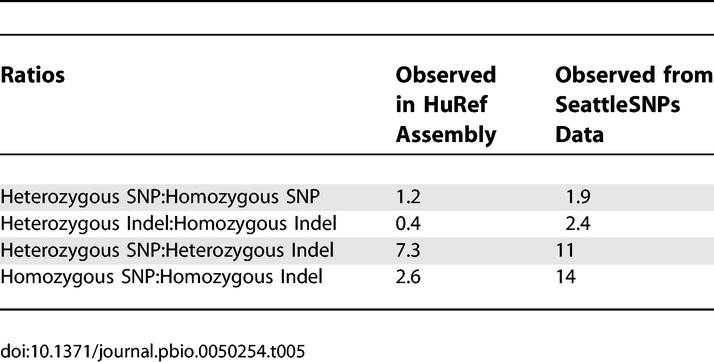
Modeling the Occurrence of Heterozygous to Homozygous Variant in a Shotgun Assembly

We also modeled the inter/intraindividual genome comparison using directed resequencing data from SeattleSNPs data (see Materials and Methods) to determine if our variant detection frequencies were commonly found for different types of variants. By sampling and comparing the genotypes of two individuals from the SeattleSNPs data, we were able to simulate the conditions for calling “heterozygous” and “homozygous” variants as we have defined them in an independently generated set (Table 5). The ratio of heterozygous variants to homozygous variants from the modeled SeattleSNPs is lower in the HuRef genome compared with the SeattleSNPs data. This suggests that there are an overabundance of homozygous variants and/or an under-representation of heterozygous variants, and this trend is more pronounced for indels compared to SNPs. A possible explanation for this is that homozygous genotypes are actually heterozygous and the second allele is missed due to low sequence coverage. Our attempts to explain this phenomenon using statistical modeling did support our hypothesis that low sequence coverage resulted in excess homozygous over heterozygous variant calls. Indeed, our modeling provided us with a bound on the missed heterozygous calls for both indels (described below) and SNPs (see section below titled: Experimental Validation of SNP Variants).

In an attempt to explain the discrepancy in the heterozygous to homozygous indel ratio (Table 5), we modeled the rate of identification of true heterozygous variants given the depth of coverage of HuRef sequencing reads and the various variant filtering criteria. This enabled us to determine that between 44% and 52% of the time, heterozygous indels will be missed due to insufficient read coverage at 7.5-fold redundancy and these indels be erroneously called homozygous. Therefore, the projection for the true number of homozygous indels is between 418,731 and 459,639, a reduction of 17%–25% from the original number of 559,473 homozygous indels, and the corresponding ratio of heterozygous to homozygous indels is between 1:1 and 1.3:1. Furthermore, our modeling also allowed us to determine that approximately 20× sequence coverage would be required to detect a heterozygous variant with 99% probability in unique sequence given our current filtering criteria of random shotgun sequence reads.

Another further explanation for the overabundance of homozygous indels is the error-prone nature of repeat regions. Using a subset of genes (55) completely sequenced by SeattleSNPs, we found that 28% of the potential 92 HuRef homozygous indels overlap with indels in these genes, as opposed to 75% confirmation rate for homozygous SNPs described earlier. When one categorizes the repeat status of a homozygous indel, a higher confirmation rate (46%) is seen for indels excluded from regions identified by RepeatMasker or TandemRepeatFinder. The confirmation rate for an indel in a transposon or tandem repeat region is much lower at 16%. Therefore, indels in nonrepetitive loci have a higher probability of authenticity than indels in repeat regions.

The ratio of SNPs to indels is lower in the HuRef assembly than what is observed by the SeattleSNPs data (Table 5), indicating that relatively fewer SNPs or relatively more indels are called. This is likely due to relatively more indels being identified, as discussed above. We note that a large fraction of indels occur in repeat sequence (Table 6), which has higher indel frequency as well as higher incidence of sequencing error. Moreover, SeattleSNPs resequencing data is focused on variant discovery in genic regions, which may not reflect genome-wide indel rates.

**Table 6:**
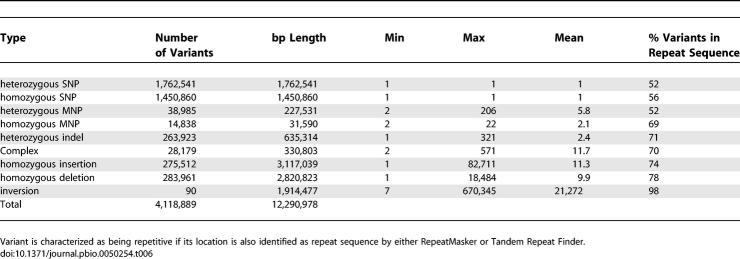
Summary of Variant Types Identified in the HuRef Genome Assembly

**Figure 6:**
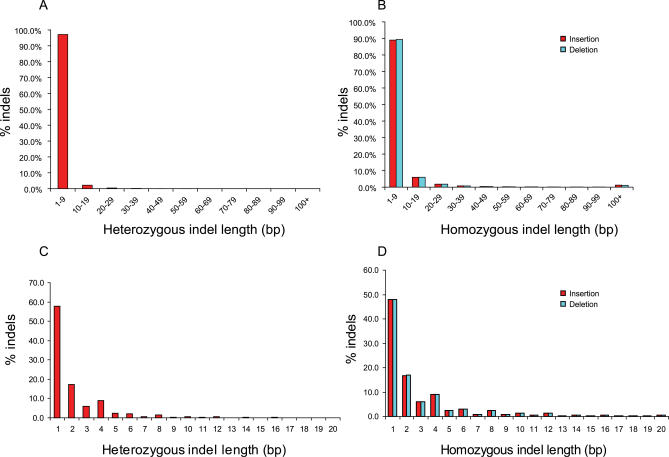
Distribution of Indel Length in the HuRef Genome Distributions of heterozygous (A) and homozygous (B) indels lengths of 1–100 bp (A and B, respectively) and at greater detail in the range 1–20 bp (C and D, respectively). Note that heterozygous indels range from 1–321 bp and homozygous indels between 1–82,711 bp, however both polymorphisms type have greater than 47% of indel events being single base. Also even-length indels appear to be overrepresented.

We identified in the HuRef assembly 263,923 heterozygous indels spanning 635,314 bp, with size ranges from 1 to 321 bp. The characteristics of the indels we detected, their distribution of sizes <5 bp, and the inverse relationship of the number of indels to length are similar to previous observations [50,51] (Figure 6A and 6B). As noted previously (Table 6), there are 2-fold more homozygous indels (559,473) than heterozygous indels, and these span 5.9 Mb and range from 1 to 82,771 bp in length. We observe that genome-wide, even-length indels are more frequent than odd-length indels (Figure 6C and 6D, χ^2^ = 12.4; *p* < 0.001, see Materials and Methods). One possible explanation for these results is that tandem repeats often have motif sizes that occur in even numbers, such as through the expansion of dinucleotide repeats. In fact, based on RepeatMasker, the majority of simple repeats are composed of even-numbered–sized motifs rather than odd-numbered–sized motifs (73%). Furthermore, of the heterozygous indels that occur in simple repeats identified by RepeatMasker, 79% occur in even-numbered bp repeats. This suggests that the preponderance of even-base–sized indels likely results from the inherent composition of simple repeats.

**Figure 7:**
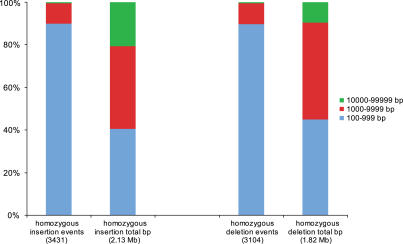
Number and Length Distribution of Apparent Homozygous Insertion and Deletion Sequences Greater than 100 bp Note that the number of indel events are similar but that there are more longer insertions than deletions.

There are 6,535 homozygous indels that are at least 100 bases in length for which both flanks of the indel can be located precisely on HuRef and NCBI assemblies. These comprise 3,431 insertions uniquely occurring on HuRef, totaling 2.13 Mb, and 3,104 deletions, totaling 1.82 Mb, found only on NCBI (Figure 7). These homozygous indels have a higher representation of repetitive elements (66%–67%) than the overall HuRef and NCBI assemblies (each 49%). This enrichment derives mainly from a higher relative content of short interspersed nuclear elements (SINEs), simple repeats, and unclassified SVAs (Table 7). For 657 (19% of the total) insertions with a minimum length of 100 bp, at least 50% of the segment length (mean = 95%) is composed of a single SINE insertion. Most of these SINE insertions (88%) belong to the youngest Alu family (AluY), for which insertion polymorphisms are well documented in the human genome [52,53]. Similarly, for 26% of deletions at least 100 bp in length, an average of 95% of the segment consists of a single SINE element, and 92% of these elements are classified as AluY. Interestingly, the combined total of 1,316 AluY insertions that differ between HuRef and NCBI include 703 (53%) that are not currently identified in the most comprehensive database of human bimorphic SINE insertions, the database of retrotransposon insertion polymorphisms in human (dbRIP;1625 loci; http://falcon.roswellpark.org:9090/) (Table S4) [54].

**Table 7:**
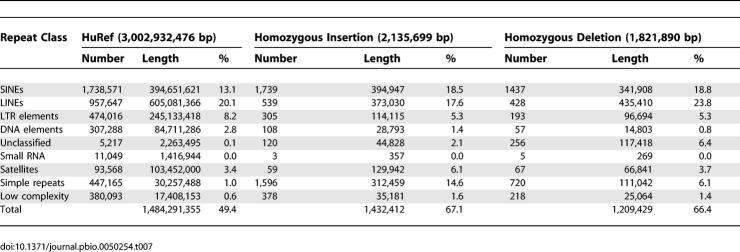
Repetitive Elements in the Complete HuRef Assembly, Homozygous Insertions and Deletions Were Identified Using RepeatMasker

### Experimental Validation of SNP Variants

**Figure 8:**
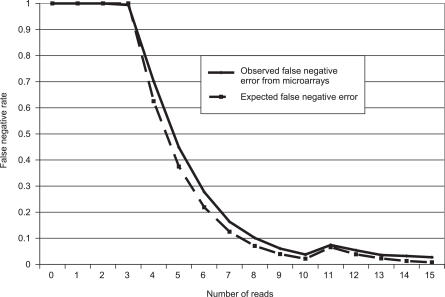
Modeling the Rate of SNP Detection from Microarray Experiments Model of the false-negative rate of heterozygous SNP detection found on Affymetrix or Illumina genotyping platforms in relation to the number of supporting reads found in the HuRef assembly at these loci. The observed false-negative rate of detected heterozygous SNPs in the HuRef assembly closely follows the modeled rate given a Poisson model. The predicted false-negative error is based on the thresholds of requiring at least 20% of the reads supporting the minor allele, two reads minimum. The increased false-negative error at 11 is due to the increased number of reads required to call the minor allele compared to two reads being required at 4×–10× coverage. Therefore, at 11×–15× coverage, three reads are required. The additional read changes the binomial distribution and increases false-negative error (See Materials and Methods).

**Figure 9:**
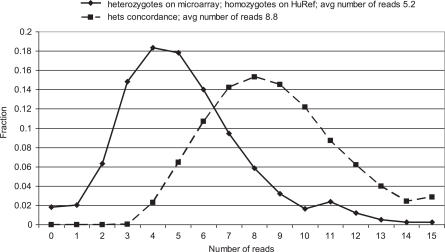
Distribution of HuRef Read-Depth Coverage for Genotyped SNPs Distribution plot of number of underlying reads (average number of reads = 8.8) in HuRef heterozygous SNPs confirmed by the Affymetrix and Illumina genotyping platforms. This is compared to a distribution (average number of reads = 5.2) for SNP detected by the platforms but missed in the HuRef assembly.

To evaluate the accuracy and validity of SNP calling from the sequencing reads, the donor DNA was interrogated using hybridization-based SNP microarrays: the Affymetrix Mapping 500K Array Set, which targets 500,566 SNP markers, and the Illumina HumanHap650Y Genotyping BeadChip, which targets 655,362 SNPs. The Affymetrix array experiment was performed twice to provide a technical replicate for genotyping error estimation, and 0.12% of genotype calls were discordant. Of the 92,144 assays with an annotation in dbSNP that overlap between the two different platforms, 99.87% were concordant (0.13% discordant). Thus, the discordance rate between platforms was similar to that between Affymetrix technical replicates. Genotype calls that were discordant between technical replicates or between the Affymetrix and Illumina platforms were excluded from further analysis. This resulted in 1,029,688 nonredundant SNP calls from the two genotyping platforms, which were then compared to the HuRef assembly and to the single nucleotide variants extracted from the sequencing data. Of these, 943,531 genotypes (91.63%) were concordant between the genotyping platforms and the HuRef assembly (Table 8). Of the 86,157 discordant genotype calls, the vast majority (83.9%) were identified as heterozygous in the merged genotyping platform data, but called as homozygous in the HuRef assembly (Table 9). This is consistent with a predictable effect of finite sequence coverage in the HuRef dataset: assuming uniform random sampling of both haplotypes, 21.6% of true heterozygous SNPs are expected to be missed given 7.5× coverage of the diploid genome and the requirements for calling a heterozygous SNP (i.e., at least two instances of each allele and ≥20% of reads confirming the minor allele). This is close to the observed false-negative error of 24.6% (Table 9 and Figure 8). Consistent with this explanation, the level of coverage is significantly lower for the missed heterozygous SNPs than for the heterozygous SNPs detected in the HuRef assembly (average read depth 5.2 and 8.8, respectively) (Figure 9).

**Table 8:**
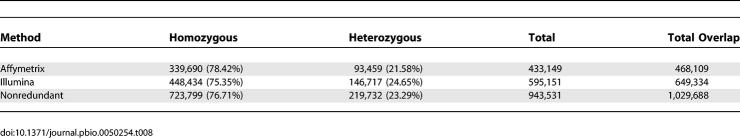
Concordancy in SNP Genotyping Validation Comparing Independent Genotype Calls Using Affymetrix 500K, Illumina HumanHap650Y in Comparison with Sequence from the HuRef Assembly

**Table 9:**
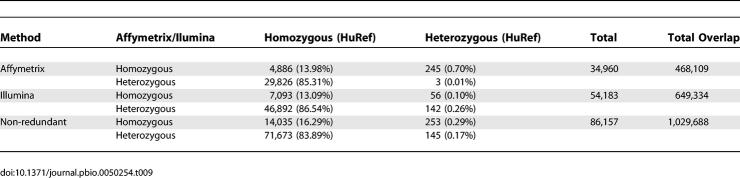
Discordant Calls in SNP Genotyping Validation Using Affymetrix 500K, Illumina HumanHap650Y in Comparison with Sequence from the HuRef Assembly

Another possible form of error would be to erroneously call a truly homozygous position a heterozygous variant. Of the 65,337 homozygote calls that were concordant between the Affymetrix and Illumina platforms, none were called as heterozygous in the HuRef assembly. Therefore, the upper bound for the false-positive rate is 0.0046% (one-tailed 95% confidence interval), and one would expect false-positive heterozygote calls approximately once every 22 kb from the upper bound of this confidence interval. However, this estimate may be lower than the genome-wide false-positive error, because it is based on the positions chosen by the microarray platforms, which tend to be biased away from repetitive, duplicated, and homopolymeric regions. Approximately three-quarters of the novel heterozygous SNPs (73%) and novel heterozygous indels (75%) are in a region identified by RepeatMasker, TandemRepeatFinder, or a segmental duplication. Therefore, approximately three-quarters of the novel heterozygous variants are in regions that are most likely underrepresented in the microarrays. Consequently, we cannot readily extrapolate the false-positive error determined from the microarrays to be the discovery rate of the HuRef variant set. The repetitive regions are likely to have a higher false-positive rate due to sequencing error and misassembly. Further, they are not represented in the current estimate of the false-positive rate. However, they also exhibit a higher rate of authentic variation.

### Computational Validation of Indels

Homozygous and heterozygous insertions and deletions identified in the HuRef assembly were computationally validated by comparison to previously published datasets. As indicated in Figure 4, the homozygous insertion and deletions variants are operationally defined as either inserted or deleted sequence in the HuRef genome respectively since there is no other read evidence for heterozygosity. The homozygous nature of these variants does not imply any notion of ancestral allele. The largest set of indel variants that has been published is based on mapping of trace reads to the NCBI human genome reference assembly [55]. This approach can be used to identify deletions of any size and insertions that are small enough to be spanned by sequence reads. In this analysis, the 216,179 deletions and 177,320 insertions from Mills et al. [55] were compared to the insertions and deletions identified from the HuRef assembly. Based on this analysis, we found support for 37,893 homozygous deletions and 46,043 homozygous insertions that overlapped between the two datasets (Table 11). Comparison with the heterozygous deletions and insertions from the HuRef assembly yielded support for 9,431 deletions and 7,738 insertions, respectively (Table 10). These values represent a lower limit due to possible alignment issues in regions with tandem repeats. This dataset produced the largest overlap with the HuRef variant set compared to all others discussed below. However the Mills et al. published dataset used reads from the NCBI TraceArchive that we also used during assembly (i.e., Celera reads, donor HuBB). This suggests that essentially the same dataset used by two different groups produced an overlapping result by using different methods. As a consequence, we cannot determine which part of the overlapped variants with the Mills et al. data came from non-Celera sources, and therefore we cannot comment on novelty or polymorphic supporting evidence for HuRef variants.

**Table 10:**
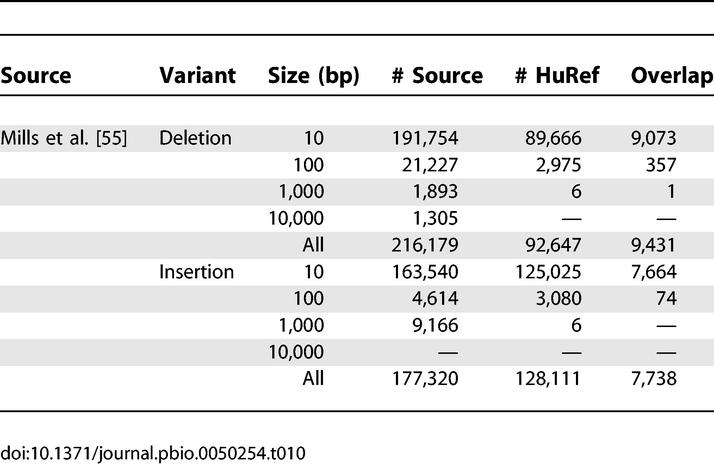
Comparison of HuRef Heterozygous Indels to Indel Variants Identified from Other Studies

**Table 11:**
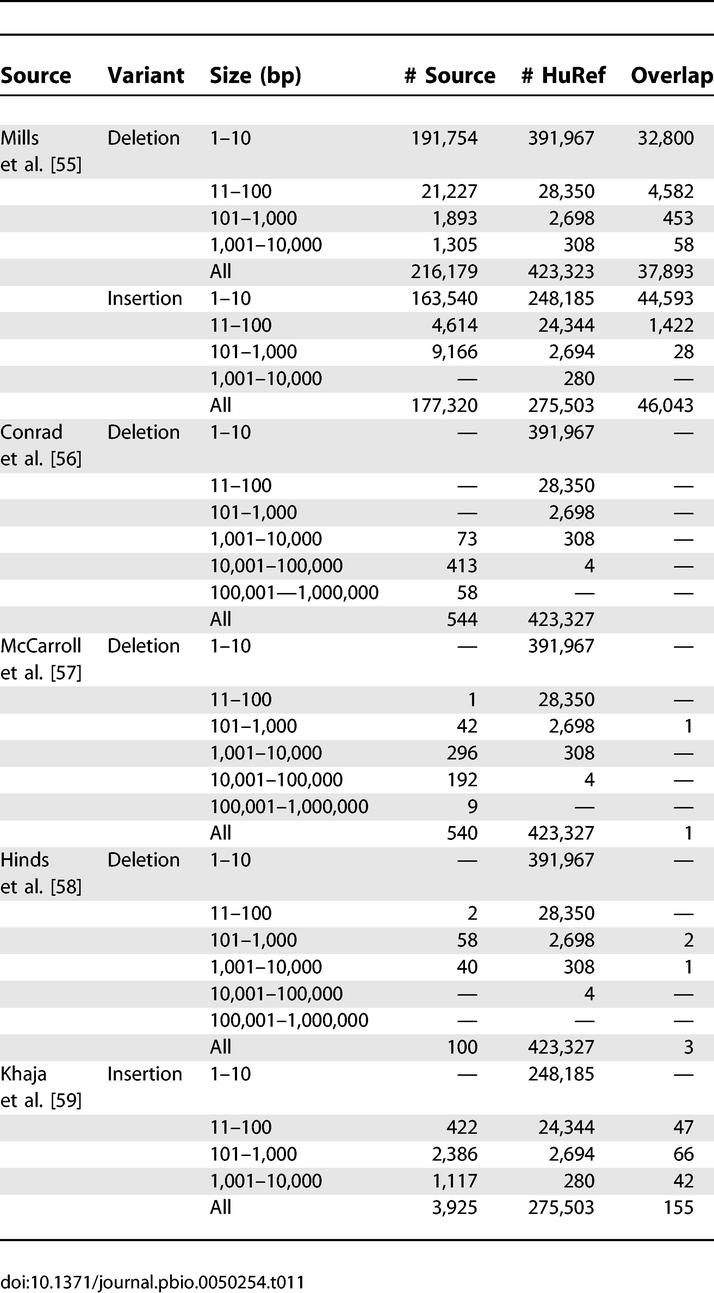
Comparison of HuRef Homozygous Indels to Indel Variants Identified from Other Studies

Next, the HuRef homozygous deletions were compared to three other sets of previously identified deletion polymorphisms [56–58]. However, the overlap with these datasets was minimal, possibly due to the larger size of these variants (Table 11). Finally, the set of HuRef homozygous insertions was compared to those variants identified in an assembly comparison approach [59], and support was found for additional 243 insertion variants.

We sought further evidence in support of the longest indels identified by the one-to-one HuRef–NCBI mapping. We focused on the 20 longest insertions (9–83 kb) and the 20 longest deletions (7–20 kb) and examined the presence of these large indels in the genomes of eight other individuals by identifying fosmid clones that map to these 40 loci (Table S5). The fosmid mapping provided support for all 20 insertions, and 17 of 20 deletions. The lack of support for two of the deletions (Unique Identifiers 1104685056026, 1104685093410) is likely due to their location at the ends of HuRef scaffolds, which greatly reduces the possibility of mapping fosmids that span the insertion site. Support from multiple fosmids provides the strongest evidence for variation in indels between individuals. For example, the presence of a 24 kb insertion on Chromosome 22 (Unique Identifier 1104685552590) is supported by 13–17 fosmids in three individuals (with no evidence for absence), whereas its absence is supported by 19 fosmids in another individual (with no evidence for presence). These data suggest that the majority of large indels defined by the one-to-one HuRef–NCBI mapping are genuine variations among human genomes.

### Experimental Verification of Heterozygous Indel Variants

We selected 19 non-genic heterozygous indels in a nonrandom manner, ranging in length from 1 to 16 bp, for experimental validation using PCR coupled with PAGE detection of allelic forms. We ensured that the read depth coverage was in an acceptable range (not greater than 15 reads), suggesting that these loci were not in segmental duplications and would therefore not produce spurious PCR amplification. Three Coriell DNA samples and HuRef donor DNA were examined, and 15 out of 19 PCR assays assessed generated results consistent with the positive and negative controls. The indel lengths that yielded experimental data ranged from 1 to 8 bp in length. In four out of 15 indels, the heterozygote variant was identified in all four DNA samples, and in three out of 15, it was only found the HuRef donor DNA. For the remaining eight out of 15 cases, the indels were differentially observed among the four DNA samples (Figure S1).

### Experimental Verification of Characterized Homozygous Insertion/Deletion Variants

We selected 51 putative homozygous HuRef insertions in a nonrandom manner for validation in 93 Coriell DNA samples based on their proximity to annotated genes, their size range of 100–1,000 bp, the absence of transposon repeat or tandem repeat sequence, uniqueness in the HuRef genome, and the absence of any similarity to chimpanzee sequence. The experimental results (Table S6) indicated that for 43 of 51 insertions (84%), we were able to generate specific PCR products for which the size of PCR products were as predicted and fell within the detectable range of the gel. For 84% of these 43 cases, insertions were identified in HuRef and additional DNA samples, and most follow Hardy-Weinberg equilibrium in CEU samples. Approximately 7% of the insertions tested (3 of 43) were false positives, because the HuRef donor DNA and all the 93 Coriell DNAs were homozygous for no insertion. In four insertions (9%), all of the tested Coriell samples displayed normal Hardy-Weinberg equilibrium; however, the insertion was absent in the HuRef sample. The inability to observe the insertion in the HuRef sample in these instances might be due to allelic dropout in the PCR process for the HuRef sample. This could be caused by specific SNPs at the primer annealing sites that were not accounted for during the primer design process.

In 22 (61%) confirmed experiments, the HuRef donor bears homozygous insertions in agreement with our computational analyses. There are four insertions in this set, among the 22, where the HuRef donor and all 93 Coriell DNA donors tested were homozygous for insertions. This suggests that these sequences were either not assembled in the NCBI human genome assembly or that the NCBI donor DNA sequenced had a rare deletion in these regions.

For the remaining 14 insertions (39%), the HuRef donor was heterozygous for the insertion instead of homozygous as was predicted by our indel detection pipeline. We searched for these alternative shorter alleles in the HuRef assembly and observed that two of the alternative alleles matched degenerate scaffolds and two matched singleton unassembled reads. These are sequence elements that are typically small or unassembled elements respectively, signifying that the assembly process selected one allele.

We note that many of the insertions tested (84%) are polymorphic in the Coriell panel tested, and although many are intronic, there are instances of UTR and exonic insertions whose impact on function may be more directly ascertained.

### Analysis of Segmental Duplications

It has previously been shown that extended regions of high sequence identity complicate de novo genome assembly [10,60,61]. An analysis was undertaken to assess how well the segmental duplications (identified as regions of >5 kb with >90% sequence identity) annotated in the NCBI assembly are represented in the HuRef genome sequence. We analyzed the NCBI sequence (90.1 Mb) external to the one-to-one mapping with the NCBI assembly for segmental duplication content by comparison to the Human Segmental Duplication Database (http://projects.tcag.ca/humandup/) [61]. More than 70% of these nucleotides (63.6 Mb) are contained within segmental duplications, compared with 5.14% across the entire NCBI assembly. This suggests that the regions of the NCBI assembly that are not aligned to HuRef likely result from the absence of assembled segmental duplication regions in HuRef. This is further supported by the fact that only 57.2% of all regions annotated as segmental duplications in NCBI are present in HuRef. Clearly, these are some of the most difficult regions of the genome to represent accurately with a random shotgun approach and de novo assembly. However, it is also important to note that at least 25% of segmental duplication regions differ in copy number between individuals [62], and the annotation of such sequences will certainly differ between independent genomes.

### Copy Number Variants

Copy number variants (CNVs) have been identified to be a common feature in the human genome [11,15,62–64]. However, such variants can be difficult to identify and assemble from sequence data alone, because they are often associated with the repetition of large segments of identical or nearly identical sequences. We tested for CNVs experimentally to compare against those annotated computationally, and also to discover others not represented in the HuRef assembly. We used comparative genomic hybridization (CGH) with the Agilent 244K array and Nimblegen 385K array, as well as comparative intensity data from the Affymetrix and Illumina SNP genotyping platforms (using three analysis tools for Affymetrix and one for Illumina). In total, 62 CNVs (32 losses and 30 gains) were identified from these experiments (Table S7). It is noteworthy that the Agilent and Nimblegen CGH experiments, as well as the analysis of Affymetrix data using the GEMCA algorithm, were run against a single reference sample (NA10851). Therefore, a subset of the regions reported as variant may reflect the reference sample rather than the HuRef donor, even though all previously identified variants in the reference sample [62] were removed from the final list of CNV calls in the present study. The majority of the variant regions were detected by only one platform, reflecting the difference in probe coverage and sensitivity among various approaches [12,62]. As an independent form of validation, the CNVs detected here were compared to those reported in the Database of Genomic Variants (DGV) [63], and 54 of the variants (87%) have been described previously (with the thresholds used for these analyses we expect approximately 5% of calls to be false positive). A summary of the genomic features overlapped by these CNVs is presented in Table 12. Approximately 55% of the CNVs overlap with annotated segmental duplications, which is slightly higher than reported in previous studies [63,64]. The CNVs also overlap 95 RefSeq genes, seven of which are described in the Online Mendelian Inheritance in Man database (OMIM) as linked to a specific phenotype (Table S7). These include blood group determinants such as RHD and XG, as well as a gain overlapping the coagulation factor VIII gene.

**Table 12:**
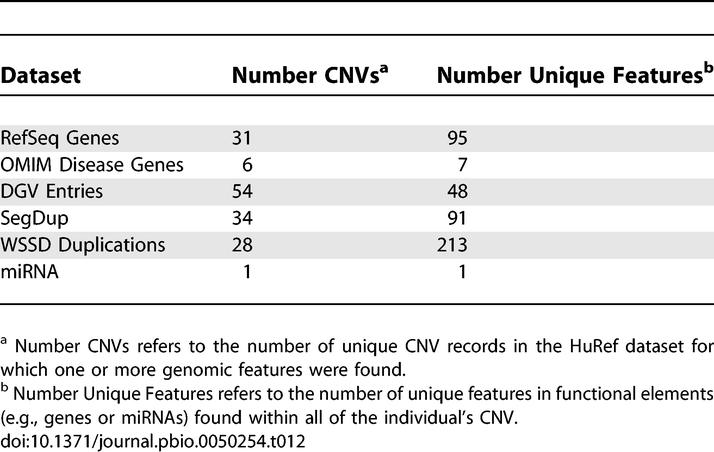
Copy Number Variants Identified on the HuRef Sample

### FISH of Unmapped HuRef Scaffolds

**Figure 10:**
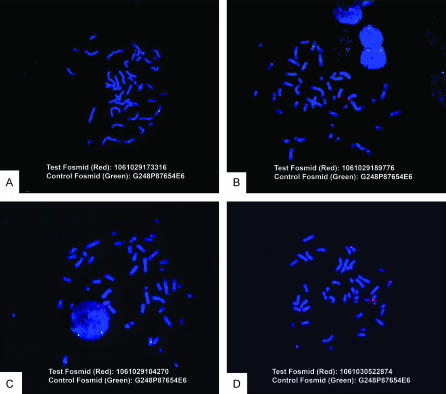
Non-Mapped HuRef Sequences Mapped to Coriell DNA Samples by FISH Sequences from the HuRef donor that had no match based on the one-to-one mapping or BLAST when compared to the NCBI Human reference genome were tested by FISH. Fosmids were used as probes and the experiments were run, using Coriell DNA, to confirm the localization of the contigs or to map contigs with no prior mapping information. Shown here are four representative results. (A) An insertion at 7q22 where the FISH confirmed the HuRef mapping, (B) FISH result confirming the mapping of a sequence extending into a gap at 1p21. (C) Localization of a contig with no prior mapping information to chromosomal band 1q42. (D) An example of euchromatic-like sequence with no prior mapping information, which hybridizes to multiple centromeric locations.

Numerous HuRef sequences that span the entire or partial scaffolds did not have a matching sequence in the NCBI genome. Some had putative chromosomal location assignments (e.g., sequences extending into NCBI gaps), whereas others were unanchored scaffolds with no mapping information. We selected sequences >40 kb in length with no match to the NCBI genome and identified fosmids (derived from the Coriell DNA NA18552) mapping to these sequences based clone end-sequence data. The fosmids were then used as FISH probes with the aim of confirming annotated locations for anchored sequences and assigning chromosomal locations to unanchored scaffolds. Fosmids were hybridized to metaphase spreads from two different cells lines. At least 10 metaphases were scored for each probe, and a differentially labeled control fosmid was included for each hybridization. For 23 regions, there was no mapping information available from mate-pair data or the one-to-one mapping comparison. Of the remaining 26 regions, 24 had a specific chromosomal location assigned at the nucleotide level (Figure 10A and 10B), whereas two regions were assigned to specific chromosomes but lacked detailed mapping information. The results of the FISH experiments are outlined in Table S8. Of the 23 regions with no prior mapping information, 13 gave a single primary mapping location (Figure 10C). The majority of the remaining 10 regions located to multiple centromeric regions (Figure 10D), suggesting that there are large euchromatic-like sequences present as low-copy repeats in the current centromeric assembly gaps. For the 26 regions with mapping information, the expected signal was observed for 22 (85%). However, in six of these hybridizations, there were additional signals of equal intensity at other locations. Ten of the scaffolds chosen for FISH extend into contig or clone gaps in the current reference assembly. Of these 10 regions, the expected localization was corroborated for seven. The combined data indicate that the HuRef assembly contributes significant amounts of novel sequence important for generating more complete reference assemblies.

### Haplotype Assembly

Haplotypes have more power than individual variants in the context of association studies and predicting disease risk [65–67] and also permit the selection of reduced sets of “tagging” SNPs, where linkage disequilibrium is strong enough to make groups of SNPs largely redundant [68,69]. The potential for shotgun sequences from a single individual to be used to separate haplotypes has been examined previously [70,71]. For a given polymorphic site, sequencing reads spanning that variant can be separated based on the allele they contain. For data from a single individual, this amounts to separation based on chromosome of origin. When two or more variant positions are spanned by a single read, or occur on paired reads derived from the same shotgun clone, alleles can be linked to identify larger haplotypes. This is sometimes known as “haplotype assembly.” When single shotgun reads are considered, the problem is computationally tractable [70,71] but the resulting partial haplotypes would be quite short with reads produced by existing sequencing technology, given the observed density of polymorphisms in the human genome (R. Lippert, personal communication). Mate pairing has the potential to increase the degree of “haplotype assembly,” but finding the optimal solution in the presence of errors in the data has been shown to be computationally intractable [71]. Nevertheless, we show that the character and quality of the data is such that heuristic solutions, while not guaranteed to find the best possible solution, can provide long, high-quality phasing of heterozygous variants.

**Figure 11:**
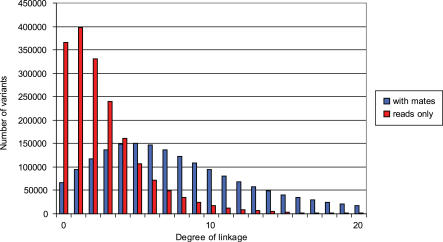
Degree of Linkage of Heterozygous Variants The distribution of the number of other variants to which a given variant can be linked using sequencing reads only or using mated reads as well is shown. Linkage of variants based on individual sequencing reads is limited, regardless of sequence coverage beyond a modest level, but is substantially increased by the incorporation of mate pairing information. The size of the effect is considerably more than simply doubling read length, due to variation in insert size; consequently, benefits of increasing sequencing coverage drop off much more slowly.

The set of autosomal heterozygous variants described above (*n* = 1,856,446) was used for haplotype assembly. The average separation of these variants on the genome was ∼1500 bp (twice the average read length). Fewer than 50% of variants could be placed in “chains” of six or more variants where successive variants were within 1 kb of one another. Consequently, single reads cannot connect these variants into large haplotypes. However, the effect of mate pairing is substantially greater than would be observed simply by doubling the length of a read, as shown in Figure 11: variants are linked to an average of 8.7 other variants.

**Figure 12:**
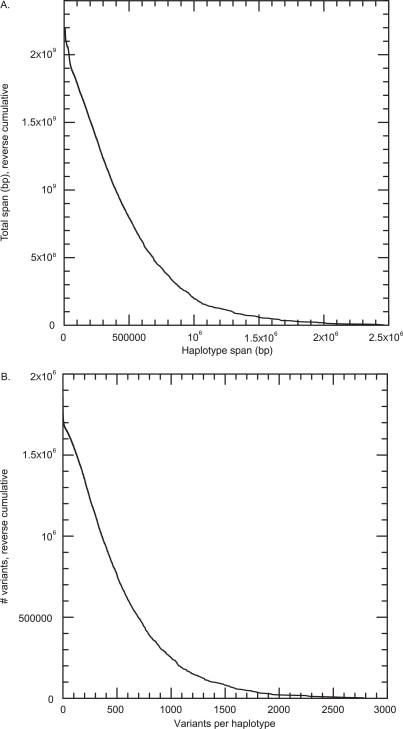
Distribution of Inferred Haplotype Sizes (A) Reverse cumulative distribution of haplotype spans (bp) (N50 ∼ 350 kb). (B) Reverse cumulative distribution of variants per haplotype (N50 ∼ 400 variants).

Using this dataset, haplotype assembly was performed as described in Materials and Methods. Half of the variants were assembled into haplotypes of at least 401 variants, and haplotypes spanning >200 kb cover 1.5 Gb of genome sequence. The full distributions of haplotype sizes, both in terms of bases spanned and in terms of numbers of variants per haplotype, are shown in Figure 12 *.* Although haplotypes inferred in this fashion are not necessarily composed of continuous variants, haplotypes do in fact contain 91% of the variants they span. More than 75% of the total autosomal chromosome length is in haplotypes spanning at least four variants, and 89% of the variants are in haplotypes that include at least four heterozygous HapMap (phase I) variants.

Both internal consistency checks and comparison to HapMap data indicate that the HuRef haplotypes are highly accurate. Comparing individual clones against the haplotypes to which they are assigned, 97.4% of variant calls were consistent with the assigned haplotype. Moreover, the HuRef haplotypes were strongly consistent with those inferred as part of the HapMap project [35]. Where a pair of variants is in strong LD according to the HapMap haplotypes, the correct phasing of the HuRef data would be expected to match the more frequent phasing in the HapMap set in most cases. Exceptions would require a rare recombination event, convergent mutation in the HuRef genome, or an error in the HapMap phasing in multiple individuals.

**Figure 13:**
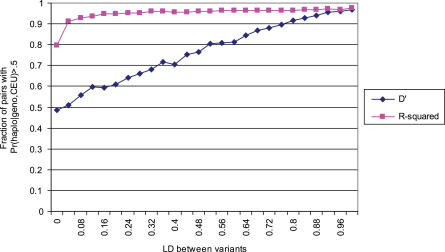
Consistency of HuRef Haplotypes with HapMap Data Haplotypes inferred from the HuRef data are strongly consistent with HapMap haplotypes. The probability in the HapMap CEU panel of the observed genotypes being phased as per the HuRef haplotypes is high for variants in strong LD (as measured either by D′ or *r* ^2^)*.*

We accessed the 120 phased CEU haplotypes from HapMap and identified the subset of heterozygous HuRef SNP variants that also coincided with the HapMap data. For adjacent pairs of such variants that were in strong LD (*r* ^2^ ≥ 0.9; *n =* 197,035), fewer than 1 in 40 of the HuRef-inferred haplotypes conflicted with the preferred HapMap phasing. Figure 13 shows more generally the consistency of HuRef haplotypes with the HapMap population data as a function of *r* ^2^ and D′. Because the inference of HuRef haplotypes is completely independent of the data and methods used to infer HapMap haplotypes, this is a remarkable confirmation of the HuRef haplotypes.

The restriction to variants in strong LD has no clear selection bias with respect to our inferred haplotypes. On the other hand, it provides only weaker confirmation for the HapMap phasing, since it is restricted to the easiest cases for phasing using population data—namely only those pairs of variants in strong linkage disequilibrium.

The lengths and densities of the inferred HuRef haplotypes described above are possible due to the use of paired end reads from a variety of insert sizes. Given the relatively simple means that were used for separating haplotypes, the high accuracy of phasing is likewise due to the quality of the underlying sequence data, the genome assembly, and the set of identified variants. The rate of conflict with HapMap with regard to variants in high LD can be further decreased by filtering the variants more aggressively (particularly excluding indels; unpublished data), although at the expense of decreasing haplotype size and density. It is also possible to improve the consistency measures described above by using more sophisticated methods for haplotype separation. One possibility we have explored is to use the solutions described above as a starting point in a Markov chain Monte Carlo (MCMC) algorithm. This produces solutions for which the fraction of high LD conflicts with HapMap is reduced by ∼30%. This approach has other advantages as well: MCMC sampling provides a natural way to assess the confidence of a partial haplotype assignment. Assessment of this and other measures of confidence is a topic for future investigation.

We used the generated haplotypes to view how well they span the current gene annotation. We were able to identify 84% (19,407 out of 23,224 protein coding genes) of Ensembl version 41 genes partially contained within a haplotype block and 58% of protein coding genes completely contained within a haplotype block. We note that in population-based haplotypes, denser sampling of SNPs in regions of low LD leads to reduction in the size of the average haplotype block [72]. In contrast to this finding, detection of additional true heterozygous variants through personal sequencing, regardless of LD, would lead to larger partial haplotypes, because additional variants increase the density of variants and thus their linkage to one another.

### Gene-Based Variation in HuRef

**Figure 14:**
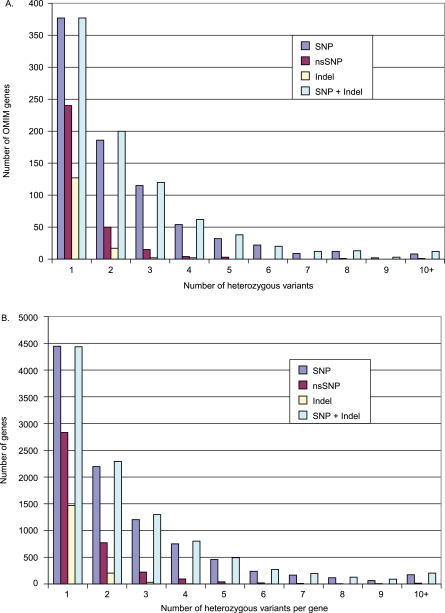
Distribution of HuRef Variants in OMIM and Ensembl Genes (A) The distribution of the OMIM genes in Ensembl version 41 protein coding genes that contain one or more SNP or indel in their coding and/or UTR regions. (B) A similar distribution for the variants found in coding and/or UTR regions for all Ensembl version 41 genes.

The sequencing, assembly, and cataloguing of the variant set and the corresponding haplotypes of the HuRef donor provided unprecedented opportunity to study gene-based variation using the vast body of scientific literature and extensively curated databases like OMIM [73] and Human Genetic Mutation Database (HGMD, [18]). A preliminary assessment indicates that 857 OMIM genes have at least one heterozygous variant in the coding or UTR regions, and 314 OMIM genes have at least one nonsynonymous SNP (Figure 14A). Overall, we observed 11,718 heterozygous and 9,434 homozygous coding SNPs and 236 heterozygous and 627 homozygous coding indels (Figure 14B). In addition, 4,107 genes have 6,114 nonsynonymous SNPs indicating that at least 17% (4,107/23,224) of genes encode differential proteins. The nonsynonymous SNPs define a lower limit of a potentially impacted proteome, because 44% of genes (10,208/23,224) have at least one heterozygous variant in the UTR or coding region and these variants could also affect protein function or expression. Therefore, almost half of the genes could have differential states in this diploid human genome, and this estimate does not include variation in nonexonic regions involved in gene regulation such as promoters and enhancers.

Understanding potential genotype-to-phenotype relationships will require many more extensive population-based studies. However, the complexities of assessing genotype–phenotype relationships begin to emerge even from a very preliminary glimpse of an individual human genome (Table 13). For Mendelian conditions such as Huntington disease (HD), the predictive nature of the genomic sequence is more definitive. Our data reveal the donor to be heterozygous (CAG)_18_/(CAG)_17_ in the polymorphic trinucleotide repeat located in the *HD* gene (HD affected individuals have more than 29 CAG repeats) [74]. The genotype matches the phenotype in this case, since the donor does not have a family history of Huntington disease and shows no sign of disease symptoms, even though he is well past the average onset age. The HuRef donor's predisposition status for multifactorial diseases is, as expected, more complicated. For example, the donor has a family history of cardiovascular disease prompting us to consider potentially associated alleles. The HuRef donor is heterozygous for variants in the KL gene; F352V (r9536314) and C370S (rs9527025). It has previously been observed that these heterozygous alleles present a lower risk for coronary artery disease [75]. However, the donor is also homozygous for the 5A/5A in rs3025058 in the promoter of the matrix metalloproteinase-3 *(MMP3)* [76]. This genotype is associated with higher intra-arterial levels of stromelysin and has a higher risk of acute myocardial infarction. This observation highlights the forthcoming challenge toward assessing the effects of the complex interactions in the multitude of genes that drive the development and progression of phenotypes. On occasion, these variant alleles may provide either protective or deleterious effects, and the ascertainment of resulting phenotypes are based on probabilities and would need to account for impinging environmental effects.

**Table 13:**
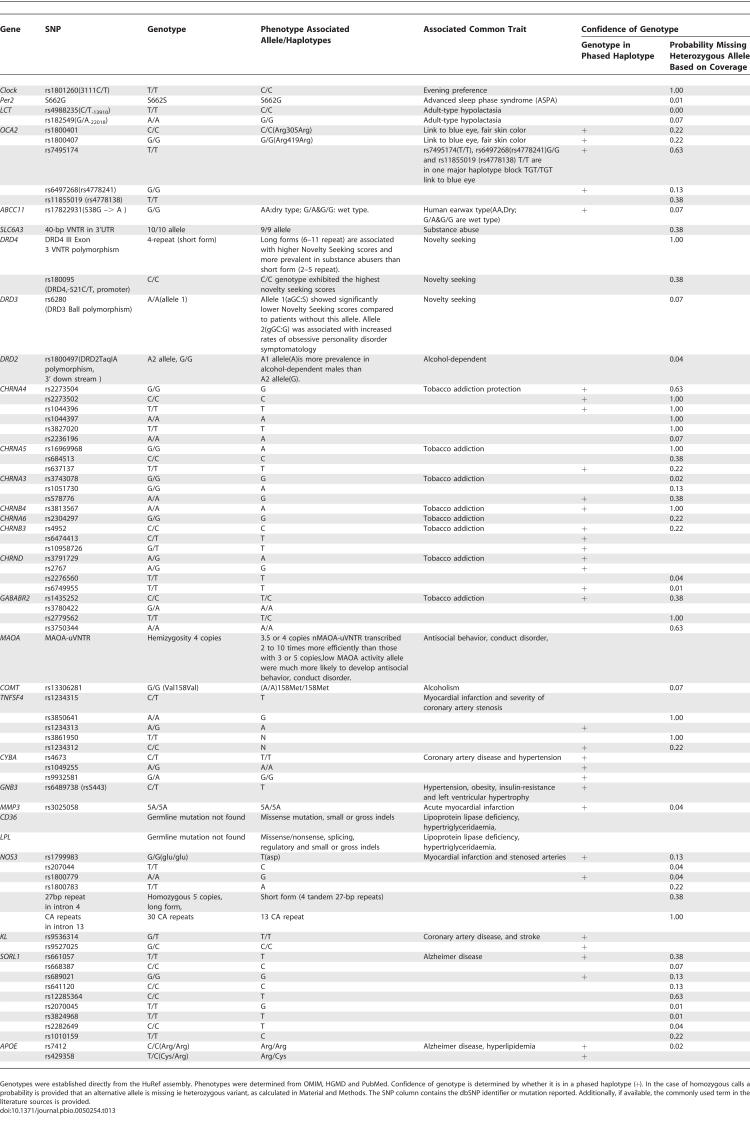
Genotypes for Some Traits in the HuRef Donor

In our preliminary analysis of the HuRef genome, we also identified some genetic changes related to known disease risks for the donor. For example, approximately 50% of the Caucasian population is heterozygous for the *GSTM1* gene, where the null mutation can increase susceptibly to environmental toxins and carcinogens [77–79]. The HuRef assembly identifies the donor to be heterozygous for the *GSTM1* gene. Currently, it is not possible without further testing (including somatic analysis) and comparison against larger datasets to determine if this variant contributes to the reported health status events experienced by the donor, such as skin cancer.

We also found some novel changes in the HuRef genome for which the biological consequences are as yet unknown. For example, we found a 4-bp novel heterozygous deletion in *Acyl-CoA Oxidase 2 (ACOX2)* causing a protein truncation. *ACOX2* encodes an enzyme activity found in peroxisomes and associates intimately with lipid metabolism and further was found to be absent from livers of patients with Zellweger syndrome [80]. The deletion identified would likely abolish peroxisome targeting, but the biological function of the mutation remains to be tested.

We have also been able to detect inconsistencies between detected genotypes in the donor's DNA and the expected phenotype based on the literature given the known phenotype of the HuRef donor. For example, the donor's *LCT* genotype should confer adult lactose tolerance according to published literature [81], but this does not match with the self-reported phenotype of the donor's lactose intolerance. Apparent inconsistencies of this nature may be explained by considering the modifying effect of other genes and their products, as well as environmental interactions.

## Discussion

We describe the sequencing, de novo assembly, and preliminary analysis of an individual diploid human genome. In the course of our study, we have developed an experimental framework that can serve as a model for the emerging field of en masse personalized genomics [82]. The components of our strategy involve: (i) sample consent and assessment, (ii) genome sequencing, (iii), genome assembly, (iv) comparative (one-to-one) mapping, (v) DNA variation detection and filtering, (vi) haplotype assembly, and (vii) annotation and interpretation of the data. We were able to construct a genome-wide representation of all DNA variants and haplotype blocks in the context of gene annotations and repeat structure identified in the HuRef donor. This provides a unique glimpse into the diploid genome of an individual human (Poster S1).

The most significant technical challenge has been to develop an assembly process (points ii–v) that faithfully maintains the integrity of the allelic contribution from an underlying set of reads originating from a diploid DNA source. As far as we know, the approach we developed is unique and is central to the identification of the large number of indels less than 400 bp in length. We attempted de novo recruitment of sequence reads to the NCBI human reference genome, using mate pairing and clone insert size to guide the accurate placement of reads [83]. Although this approach can produce useful results, it does limit variant detection to completed regions of the reference genome and, like genome assembly, can be confounded by segmentally duplicated regions.

The genome assembly approach with allelic separation allows the detection of heterozygous variants present in the individual genome with no further comparison. The one-to-one mapping of our HuRef assembly against a nearly completed reference genome permits the detection of the remaining variants. These variants arise from sequence differences found within and also outside the mapped regions, where the precision of the compared regions is being provided by the genome-to-genome comparison [59]. The ability to provide a highly confident set of DNA variants is challenging, because more than half of the variants are a single base in length but include both SNPs and indels. A filtering approach was used that accounts for the positional error profile in a Sanger sequenced electropherogram in relation to the called variant. Additional filtering considerations necessitated minimal requirements for read coverage and for the proportional representation of each allele. The filtering approaches were empirical and used the large amounts of previously described data on human variation (dbSNP). The utility of using paired-end random shotgun reads and the variant set defined on the reads via the assembly enabled the construction of long-range haplotypes. The haplotypes are remarkably well constructed given that the density of the variant map is comparable to those used in other studies [35], reflecting the utility of underlying sequence reads beyond just genome assembly. To understand how an individual genome translates into an individual transcriptome and ultimately a functional proteome, it is important to define the segregation of variants among each chromosomal copy.

While several new approaches for DNA sequencing are available or being developed [84–86], we chose to use proven Sanger sequencing technology for this HuRef project. The choice was obviously motivated in part for historical reasons [7], but not solely. We attached a high importance to generating a de novo assembly including maximizing coverage and sensitivity for detecting variation. We further anticipated that long read lengths (in excess of 800 nucleotides), compatibility with paired-end shotgun clone sequencing, and well-developed parameters for assessing sequencing accuracy would be required. High sequence accuracy is essential to avoid calling large numbers of false-positive variants on a genome-wide scale. Long paired-end reads are especially useful for achieving the best possible assembly characteristics in whole-genome shotgun sequencing and for providing sufficient linkage of variants to determine large haplotypes.

We have been able to categorize a significant amount of DNA variation in the genome of a single human. Of great interest is the fact that 44% of annotated genes have at least one, and often more, alterations within them. The vast majority—3,213,401 events (78%) of the 4.1 million variants detected in the HuRef donor—are SNPs. However, the remaining 22% of non-SNP variants constitute the vast majority, about 9 Mb or 74%, of variant bases in the donor. Using microarray-based methods, we also detected another 62 copy number variable regions in HuRef, estimated to add some 10 Mb of additional heterogeneity. Given these potential sources of measured DNA variation, we can, for the first time, make a conservative estimate that a minimum of 0.5% variation exists between two haploid genomes (all heterozygous bases, i.e., SNP, multi-nucleotide polymorphisms [MNP], indels, [complex variants + putative alternate alleles + CNV]/genome size; [2,894,929 + 939,799 + 10,000,000]/2,809,547,336) namely those that make up the diploid DNA of the HuRef assembly. We also note that there will be significantly more DNA variation discovered in heterochromatic regions of the genome [87], which largely escaped our analysis in this study.

We had mixed success when attempting to find support for the experimentally determined CNVs in the HuRef assembly itself or the data from which it was derived. More than 50% of the CNVs overlapped segmental duplications, and these regions are underrepresented in HuRef, which complicated the analysis. We attempted to map the sequence reads onto the NCBI human genome and then identify CNVs by detecting regions with significant changes in read depth. However, we found significant local fluctuations in read depth across the genome, limiting the ability for comparison and suggesting that a higher coverage of reads may be required to use this approach effectively.

As we have emphasized throughout, a major difference of the genomic assembly we have described is our approach to maintain, wherever possible, the diploid nature of the genome. This is in contrast to both the NCBI and WGSA genomes, which are each consensus sequences and, therefore, a mosaic of haplotypes that do not accurately display the relationships of variants on either of the autosomal pairs. For BAC-based genome assemblies such as the NCBI genome assembly, the mosaic fragments are generally genomic clone size (e.g., cosmid, PAC, BAC), with each clone providing contiguous sequence for only one of the two haplotypes at any given locus. Moreover, there are substantial differences in the clone composition of different chromosomes due to the historical and hierarchical mapping and sequencing strategies used to generate the NCBI reference assemblies [7,8].

In contrast, for WGSA, the reads that underlie most of the consensus sequence are derived from both haplotypes. This can result in very short-range mosaicism, where the consensus of clustered allelic differences does not actually exist in any of the underlying reads. To address this issue, the Celera assembler was modified to consider all variable bases within a given window and to group the sequence forms supporting each allele before incorporation into a consensus sequence (see Materials and Methods). In our experience, this reduces the incidence of local mosaicism, although, between windows, the consensus sequence remains a composite of haplotypes. Efforts to build haplotypes from the genome assembly (Haplotype Assembly) will likely lead to future modification of the assembler, allowing it to output longer consensus sequences for both haplotypes at many loci. Clearly, a single consensus sequence for a diploid genome, whether derived from BACs or WGS, has limitations for describing allelic variants (and specific combinations of variants) within the genome of an individual.

Partial haplotypes can be inferred for an individual from laboratory genotype data (e.g., from SNP microarrays) in conjunction with population data or genotypes of family members. However, at least in the absence of sets of related individuals (e.g., family trios), it is difficult to determine haplotypes from genotype data across regions of low LD. We have shown that sequencing with a paired-end sequencing strategy can provide highly accurate haplotype reconstruction that does not share these limitations. The assembled haplotypes are substantially larger than the blocks of SNPs in strong LD within the various populations investigated by the HapMap project. In addition to being larger, haplotypes inferred in our approach can link variants even where LD in a population is weak, and they are not restricted to those variants that have been studied in large population samples (e.g., HapMap variants). We note that in addition to the implications for human genetics, this approach could be applied to separating haplotypes of any organism of interest—without the requirement for a previous reference genome, family data, or population data—so long as polymorphism rates are high enough for an acceptable fraction of reads or mate pairs to link variants.

There are several avenues for extending our inference of haplotypes. As noted, although the naive heuristics used here give highly useful results, other approaches may give even more accurate results, as we have observed with an MCMC algorithm. There are various natural measures of confidence that can be applied to the phasing of two or more variants, including the minimum number of clones that would have to be ignored to unlink two variants, or a measure of the degrees of separation between two variants. The analysis presented here provides phasing only for sites deemed heterozygous, but data from apparently homozygous sites can be phased as well, so we can tell with confidence whether a given site is truly homozygous (i.e., the same allele is present in both haplotypes) or whether the allele at one or even both haplotypes cannot be determined, as occurs as much as 20% of the time with the current dataset. Lastly, it should be possible to combine our approach with typical genotype phasing approaches to infer even larger haplotypes.

**Figure 15:**
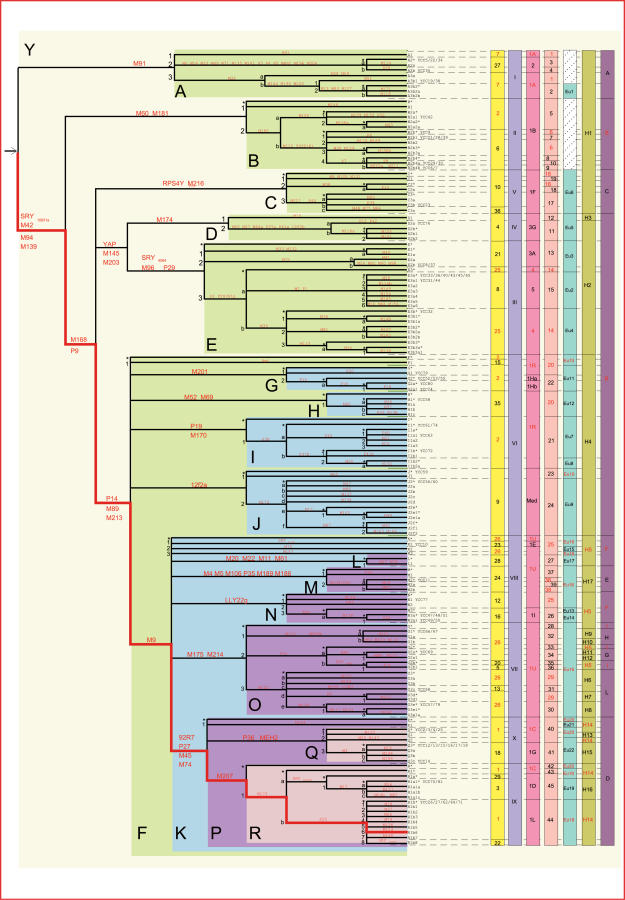
Chromosome Y ethno-genogeographic lineage The HuRef donor Y chromosome haplotype suggests descent from several European/US groups given the Y chromosome ethno-geographic markers. The haplogroup membership is R1b6 with includes individuals from the United Kingdom, Germany, Russia, and the United States, which is consistent with the self-reported family tree provided by the HuRef donor. The thick red line denotes the markers needed to trace the haplotype from the mapping of the chromosome Y markers to the HuRef genome. Data and figure from the Y Chromosome Consortium; http://ycc.biosci.arizona.edu/nomenclature_system/frontpage.html.

Our project developed over a 10-year period and the decisions regarding sample selection, techniques used, and methods of analysis were critical to the current and continued success of the project. We anticipated that beyond mere curiosity, there would be very pragmatic reasons to use a donor sample from a known consented individual. First and foremost, as we show in a preliminary analysis, genome-based correlations to phenotype can be performed. Due to the still rudimentary state of the genotype-phenotype databases it can be argued that at the present time, DNA sequence comparisons do not reveal much more information than a proper family history. Even when a disease, predisposition, or phenotypically-relevant allele is found, further familial sampling will usually be required to determine the relevance. Eventually, however, populations of genomes will be sequenced, and at some point, a critical mass will dramatically change the value of any individual initiative providing the potential for proactive rather than reactive personal health care. In a simple analogy, absent of family history, genealogical studies can now be quite accurate in reconstructing ancestral history based purely on marker-frequency comparisons to databases. Here, with a near-unlimited amount of variation data available from the HuRef assembly, we can reconstruct the chromosome Y ethno-geneographic lineage (Figure 15), which is not only consistent with, but better defines the self-reported family tree data (Figure 1A and unpublished data).

There are always issues regarding the generation and study of genetic data and these may amplify as we move from what are now primarily gene-centric studies to the new era where genome sequences become a standard form of personal information. For example, there are often concerns that individuals should not be informed of their predisposition (or fate) if there is nothing they can do about it. It is possible, however, that many of the concerns for predictive medical information will fall by the wayside as more prevention strategies, treatment options, and indeed cures become realistic. Indeed we believe that as more individuals put their genomic profiles into the public realm, effective research will be facilitated, and strategies to mitigate the untoward effects of certain genes will emerge. The cycle, in fact, should become self-propelling, and reasons to know will soon outweigh reasons to remain uninformed.

Ultimately, as more entire genome sequences and their associated personal characteristics become available, they will facilitate a new era of research into the basis of individuality. The opportunity for a better understanding of the complex interactions among genes, and between these genes and their host's personal environment will be possible using these datasets composed of many genomes. Eventually, there may be true insight into the relationships between nature and nurture, and the individual will then benefit from the contributions of the community as a whole.

## Materials and Methods

### External data sources.

We used the assembled chromosome sequence of the human genome available as NCBI version 36. The gene annotation of this genome was provided by Ensembl (http://www.ensembl.org) version 41, which incorporates dbSNP version 126. Haplotype map data was obtained from http://www.hapmap.org, Release version 21a. Celera-generated chromatograms for the HuBB individual [7] were obtained from the NCBI trace archive. These included reads from two tissues sources: blood and sperm. Sequence reads were generated from these traces using Phred version 020425.c [88] and a modified version of Paracel TraceTuner (http://sourceforge.net/projects/tracetuner/). This reprocessing significantly improved accuracy and quality in the 5′ portion of the reads, increasing their usable length by 7%, and reducing variants encoding spurious protein truncations, as well as reducing apparent heterozygous variants in the assembly.

### DNA extraction.

200-μl aliquots of thawed, whole blood were processed using the MagAttract DNA Blood Mini M48 Kit and the MagAttract DNA Blood >200 μl Blood protocol on the BioRobot M48 Workstation running the GenoM-48 QIAsoft software (version 2.0) (Qiagen; http://www.qiagen.com). Tris:EDTA (10:0.1) was used for the final 200 μl elution step. A_260_/A_280_ readings (SPECTRAmax Plus spectrophotometer (Molecular Devices; http://www.moleculardevices.com) or an ND-1000 spectrophotometer (NanoDrop Technologies; http://www.nanodrop.com), and gel images were used to quantify the DNA and to confirm that high-quality, high–molecular weight DNA was available for downstream processing. 1.0 μl of extracted DNA was run on a 0.8% agarose gel containing ethidium bromide, for 4 h at 60 V and imaged using Gel Doc and Quantity One Software (Bio-Rad Laboratories; http://www.bio-rad.com).

### Cytogenetic analysis.

Phytohemagglutin-stimulated lymphocytes from peripheral blood were cultured for 72 h with thymidine synchronization. G-banding analysis was performed on metaphase spreads from peripheral blood lymphocytes using standard cytogenetic techniques.

### Spectral karyotyping.

Spectral karyotyping was performed on metaphase spreads from cultured lymphocytes. SkyPaint probes were used according to manufacturer's instructions (Applied Spectral Imaging; http://www.spectral-imaging.com). Metaphases were viewed with a Zeiss epifluorescence microscope and spectral images were acquired with an SD300 SpectraCube system and analyzed using SkyView software 1.6.2 (Applied Spectral Imaging).

### High-throughput Applied Biosystems 3730*xl* Sanger sequencing processing.

*Plasmid and Fosmid Library Construction.* We nebulized genomic DNA to produce random fragments with a distribution of approximately 1–25 kb, end-polished these with consecutive *BAL*31 nuclease and T4 DNA polymerase treatments, and size selected using gel electrophoresis on 1% low–melting-point agarose. After ligation to *Bst*XI adapters, we purified DNA by three rounds of gel electrophoresis to remove excess adapters, inserted fragments into *Bst*XI-linearized medium-copy pBR322 plasmid vectors, and inserted the resulting library into GC10 cells by electroporation. To ensure that plasmid libraries contained few clones without inserts and no clones with chimeric inserts, we used vectors (pHOS) that include several features: (i) the sequencing primer sites immediately flank the *Bst*XI cloning site to avoid sequencing of vector DNA, (ii) there are no strong promoters oriented toward the cloning site, and (iii) *Bst*XI sites for cloning facilitate a high frequency of single inserts and rare no-insert clones. Sequencing from both ends of cloned inserts produced pairs of linked sequences of ∼800 bp each. We constructed fosmid libraries with approximately 30 μg of DNA that was sheared using bead beating and repaired by filling with dNTPs. We used a pulsed-field electrophoresis system to select for 39–40 kb fragments, which we ligated to the blunt-ended pCC1FOS vector.

*Clone Picking and Inoculation.* Libraries were propagated on large-format (16 × 16 cm) diffusion plates and colonies were picked for template preparation using a Q-bot or Q-Pix colony-picking robots (Genetix; http://www.genetix.com) and inoculated into 384-well blocks.

*DNA Template Preparation.* We prepared plasmid DNA using a robotic workstation custom built by Thermo CRS, based on the alkaline lysis miniprep [89], modified for high-throughput processing in 384-well plates. The typical yield of plasmid DNA from this method was approximately 600–800 ng per clone, providing sufficient DNA for at least four sequencing reactions per template.

*Sequencing Reactions.* Sequencing protocols were based on the di-deoxy sequencing method [90]. Two 384-well cycle-sequencing reaction plates were prepared from each plate of plasmid template DNA for opposite-end, paired-sequence reads. Sequencing reactions were completed using Big Dye Terminator (BDT) chemistry version 3.1 Cycle Sequencing Ready Reaction Kits (Applied Biosystems) and standard M13 forward and reverse primers. Reaction mixtures, thermal cycling profiles, and electrophoresis conditions were optimized to reduce volume and extend read lengths. Sequencing reactions were set-up by the Biomek FX (Beckman Coulter; http://www.beckmancoulter.com) pipetting workstations. Templates were combined with 5-μl reaction mixes consisting of deoxy- and fluorescently labeled dideoxynucleotides, DNA polymerase, sequencing primers, and reaction buffer. Bar coding and tracking promoted error-free transfer. Amplified reaction products were transferred to a 3730*xl* DNA Analyzer (Applied Biosystems).

### Genome assembly and initial variant identification.

The Celera Assembler Software (https://sourceforge.net/projects/wgs-assembler/) [7,40,91] generated contiguous sequences (contigs) that could be linked via mate-pair information into scaffolds. It has a phase for splitting initial apparently chimeric contigs (referred to as unitigs), but this process is not repeated for the final set of contigs and scaffolds as with some other assemblers (Arachne 2 [92]). This leaves a small number of chimeric scaffolds, which can be detected and split as described below. All assemblers fail to discriminate alternate alleles in polymorphic regions from distinct regions of the genome. These polymorphic regions, containing highly repetitive sequence with short unique anchoring sequence and simple algorithmic failures, result in a number of small scaffolds that are highly redundant. Although there are valuable data in these small scaffolds, they are usually not treated as part of the assembled sequence.

For this project we made specific modifications to the Celera Assembler to enable the grouping of reads into separate alleles when heterozygous variants were encountered. Instead of taking a column-by-column approach to determine the consensus sequence from a set of aligned reads, the region of variation was considered as a whole, defined as that between at least 11 bp nonvariant columns. In practice, variant regions would most frequently be single columns (SNPs), but the new algorithm only applied to longer regions. The reads spanning a variant region were split between alleles. An allele, for this purpose, was one or more spanning reads sharing an identical sequence for the variant region, and was considered confirmed if represented by two or more reads. Each allele was assigned a score equal to the sum of average quality values for the spanning portions of its reads. The highest-scoring confirmed allele was used for the consensus sequence. Alternate confirmed allele sequences were reported separately. As expected, there were usually two confirmed alleles in each region of sequence variation. Regions with more than two apparent confirmed alleles represented either collapsed repetitive sequence or a group of reads with systematic base calling error, rather than true genetic variation.

### BAC end mapping.

The set of The Institute for Genome Research (TIGR) BAC ends [41] used in the WGSA [40] assembly were aligned to the 553 HuRef scaffolds of at least 100 kb in length. We kept BAC ends that mapped uniquely to a single scaffold and near the end of a scaffold, such that their mate was likely to reside outside of the scaffold. Mate pairs were kept if both BAC ends passed the above criterion, and these indicated a possible joining of two scaffolds in a certain orientation. There were 144 consistent scaffold joins with at least two supporting mate pairs and 98 with one supporting mate pair. Using these scaffold joins would result in 409 or 311 scaffolds, respectively, of at least 100 kb, with a concomitant increase in the scaffold N50 length.

### Assembly-to-assembly mapping.

We used open-source software (http://sourceforge.net/projects/kmer/) [40,93,94] to generate a one-to-one comparison between HuRef and NCBI human genome reference assembly. For sequences that do not contain very large, nearly identical duplications, this mapping is accurate [93]. Nearly identical duplicated regions tend to be underrepresented in whole-genome shotgun assemblies such as HuRef [10]. Segments that are duplicated in one sequence but not the other (for instance when failing to merge overlapping contigs) cannot be fully included in any one-to-one mapping. For example the first few megabases of NCBI version 36 Chromosomes X and Y are identical; therefore, a 1.5-Mb scaffold from HuRef that maps to both of these regions is not part of the one-to-one mapping. Tandem repeats with variable unit copy number are also problematic for a one-to-one mapping.

For each one-to-one mapping we determined three levels: matches, runs, and clumps. A match is a maximal high-identity local alignment, usually terminated by indels or sequence gaps in one of the assemblies. Runs may include indels, and are monotonically increasing or decreasing sets of matches (linear segments of a match dot plot) with no intervening matches from other runs on either axis. Clumps are similar to runs but allow small intervening matches/runs (such as small inversions) to be skipped over. The total number of base pairs in matches is a measurement of how much of the sequence is shared between assemblies. Within a run, the number of base pairs in each assembly is different, because indels are allowed among matches in the run. These could be gaps that are filled in one assembly but not the other, polymorphic insertions or deletions, or artifactual sequence. Runs span regions in both assemblies that have no rearrangements with respect to each other, providing a direct measure of the order and orientation differences between a pair of assemblies. Clumps provide a similar measure of rearrangement but allow for small differences that may be due to noise or polymorphic inversions. Remaining sequence may be unique to one assembly or the other, but some will also be large repetitive regions without good one-to-one mapping but present in some copy number in both assemblies. Apparently unique sequence may also represent some form of contaminant.

We determined an initial set of potentially chimeric scaffolds by finding those that contained more than one clump of at least 5,000 bp relative to NCBI version 36. By mapping all HuRef and Coriell fosmid mate pairs to NCBI human reference genome and to HuRef, we assessed whether mate pair constraints were violated at the potentially chimeric junctions. Accordingly, we split 12 scaffolds.

### Variant refinement process.

DNA variants were characterized by alignment of sequencing reads in the HuRef assembly and by comparison of regions of difference in the one-to-one HuRef to NCBI reference genome map. The contribution of each sequence read to a single position in the HuRef consensus was evaluated both during and after the assembly process to identify positions that contain more than one allele. This process identified heterozygous SNPs and indel polymorphisms, and typically two or more reads were required for the initial identification of an alternate allele. Homozygous SNPs and MNPs were identified when (respectively) single or multiple contiguous loci differed in the one-to-one mapping, and all underlying HuRef reads supported one allele. Finally, homozygous insertion or deletion loci were identified where the HuRef assembly had or lacked sequence relative to the NCBI assembly, respectively. These were commonly referred to as homozygous indels unless it was relevant for analysis purposes, computational or experimental, to refer to a homozygous insertion or deletion as a way of indicating presence or absence of the sequence, respectively, in the HuRef assembly.

### Filtering of variants.

DNA variations were identified by examining the base changes within the HuRef assembly multialignment and between the HuRef assembly and the NCBI reference human genome. 5,061,599 SNPs and heterozygous variants were identified initially, after which filters were applied to eliminate erroneous calls. For a potential SNP, each read supporting that SNP was considered, and if the QV was <15 at the putative SNPs position in the read, then the read was considered invalid and was discarded as evidence for that particular variant. We also observed that deletions were overcalled at the beginnings and ends of reads, and insertions were overcalled at the ends of reads (Figure S2). By using the relative positions in the read where overcalling was detected, we were able to invalidate reads contributing to indel variant calls. We further observed that the relative read positions at which overcalling occurred was dependant on whether the read source was produced at Celera or The J. Craig Venter Institute (JCVI). Thus, any Celera read containing a putative deletion at a relative read position ≤0.18 or ≥0.76 was considered invalid for that particular deletion. Correspondingly, any JCVI read containing a putative deletion, at the relative read position ≤0.07 or ≥0.81 was deemed invalid in contributing to that particular variant call. Any Celera read was deemed invalid if it contained an insertion at a relative read position ≥0.70, and any JCVI read with an insertion at relative read position ≥0.77 was discarded as evidence. These thresholds were determined by plotting the frequency of insertions and deletions with respect to read position, and choosing the value where the call frequency was twice that of baseline (Figure S2).

Subsequent to the quality value and read location filtering the remaining variants were inspected for the percentage, number, and directionality of reads supporting the alternate alleles. Additionally these variants were inspected for the total number of reads in their assembled locus and the repeat sequence status (transposon and tandem repeat). Transposon repeats were identified using the RepeatMasker program (http://www.repeatmasker.org), and tandem repeats were identified using the Tandem RepeatFinder program [48]. The distribution of the percentage of reads containing the minor allele for heterozygous SNP and indels in Figure S3 shows that a large fraction of those putative variants that are found in dbSNP version 126 have a “minor allele frequency” (fraction of reads supporting the allele with fewer reads) of at least 20% and 25% for SNPs and indels, respectively. Therefore, we decided to apply the following filters separately to the QV and read location filtered variants, calculating at each filter step the fraction of passing variants that could be found in dbSNP. The filters applied to allow variants to be counted as bona-fide were: (i) 20% reads support minor allele for heterozygous SNP and 25% reads support minor allele for heterozygous indels, and (ii) two or more reads supporting the variant. The results of this analysis are presented in Table 3 and discussed in the Results section.

### Clustering variants.

Manual inspection showed that some neighboring variants identified within the one-to-one mapping of HuRef to the NCBI genome reference would be more precisely represented as one larger variant after realignment. To address these regions of clustered variants, we identified these problematic regions by clustering SNPs within 2 bp of each other or any non-SNP variants with 10 bp of another variant. For these variable regions, we recalled the variant(s) using the variant calling algorithm developed as part of the consensus sequence generation found in the Celera assembler.

### Filtering of homozygous insertion/deletion.

Homozygous insertion/deletions were filtered in the same manner as SNPs and heterozygous variants. All variants that were not confirmed by two or more reads were eliminated, as were those that did not fulfill minimal requirements of at least one spanning mate pair, and that the inserted sequence on the HuRef assembly or deleted sequence on the NCBI assembly not contain any ambiguous bases

### Diversity.

We estimate the population mutation parameter (θ) [43] as:

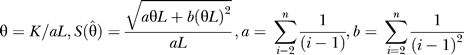
where *K* is the number of variants identified, *L* is the number of base pairs, and *n* is the number of alleles. For indels, *K* is the number of indel events. In the case of a single diploid genome, *n* = 2, so *a* and *b* reduce to 1. Then θ = *K/L,* which is simply the number of heterozygous variants divided by the length sequenced. The standard deviation of θ reduces to θ:

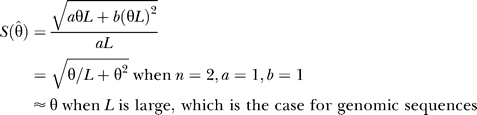
Thus, the 95% confidence interval for θ is [0, θ+2θ] or [0, 3θ].

### Estimating homozygous and heterozygous variant ratios from directed resequencing data.

Two individuals of European ancestry were randomly selected from the SeattleSNPs data (http://pga.gs.washington.edu/) [95]. For the first individual, we constructed a haploid representation (without phasing) by randomly choosing one allele at each variant position. This reconstructed sequence is analogous to the NCBI genome sequence that we used to call HuRef homozygous variants. For the second individual, all variant positions were examined and scored. If the second individual was heterozygous at a position, then the heterozygous count was incremented by one. If the second individual had a homozygous genotype that did not match the allele seen in the reconstructed sequence then the homozygous variant was incremented by one. The second individual is analogous to the HuRef assembly sequence, and this procedure mimics our variant-calling algorithm and our definitions for heterozygous and homozygous variants. One caveat is that the NCBI human genome sequence, while only being one sequence, represents multiple individuals, and thus possibly contains more rare alleles in its sequence.

### Modeling false-negative rate of heterozygous variants.

We developed a statistical model based on our assembly read coverage in the single diploid genome and on the filtering criteria used for calling high confidence variants. We assumed that chromosomes containing each of the two alleles are equally likely to be sampled and that allele loci are independent. At a given heterozygous locus, the probability of observing both alleles in at least *x* reads follows the binomial distribution with *p* = 0.50 and *n* = depth of coverage, where *x* is defined by the filtering criteria. To calculate the false-negative rate genome wide, a Poisson distribution is also incorporated to estimate sequence depth at different loci, where λ is set to the genome sequence coverage (7.5 for SNPs, 5.5 for insertions, 4.9 for deletions, after read filtering is taken into account).

### Experimental verification of heterozygous indels.

A number of heterozygous indels between 1 and 20 bp were manually selected for experimental validation by verifying trace quality in the region of the indel, read coverage depth, and repeat sequence status. In order to detect heterozygous indels from the HuRef assembly, we ran PCR-amplified genomic DNA on PAGE to look for homoduplex and heteroduplex bands. Large insertions and deletions were also recognized by this process.

Primers were designed by centering the targeted indel to produce amplicons 150–250 bp in length with the melting temperatures of these amplicons ranging between 70 °C and 86 °C. PCR for polymorphism analysis was carried out in 10-μl volume reactions containing 30 ng of purified genomic DNA, 1× PCR buffer, 20 μM deoxynucleoside triphosphates, 2 mM MgCl_2_, 8% glycerol, 0.18 μM primers, and 0.0375 U AmpliTaq Gold DNA polymerase. Post-amplification treatment of each sample involved digestion with shrimp alkaline phosphatase (0.5 U) and exonuclease I (1.76 U) for 45 min at 37 °C, 15 min at 50 °C, with heat inactivation for 15 min at 72 °C.

PAGE was carried out at room temperature for 4 h at 650 V (constant) in a standard vertical gel measuring 1 mm thick, 20 cm wide, and 30 cm long (apparatus Model SG-400–20, CBS Scientific Company Inc, http://www.cbssci.com). The native gel consisted of 10% acrylamide with the 40% acrylamide stock solution having an acrylamide/ N,N′-methylenebisacrylamide ratio of 29:1. The running buffer consisted of 1× TBE. A loading dye consisting of 2× BlueJuice (Invitrogen) was added to each amplified sample and 5 μl was loaded per gel lane. After electrophoresis, the DNA bands were visualized by staining with a 1:10,000 dilution of SYBR Gold (Invitrogen).

### Experimental validation of homozygous insertion.

Fifty-one apparent homozygous insertions in the HuRef assembly were selected based on assembly structure (appropriate read depth coverage and supporting mate pair evidence), their proximity to annotated genes, and their size. The insertion sequences were from 100 to 1,200 bp with few repeat sequences, and no detectable alignments to human (NCBI 36) or chimpanzee [22] genomes. We tested 93 Coriell DNA donors in addition to the HuRef DNA sample: 21 samples of European origin (CEU - NA06985, NA07056, NA11832, NA11839, NA11840, NA11881, NA11882, NA11992, NA11993, NA11994, NA11995, NA12057, NA12156, NA12239, NA12750, NA12751, NA12813, NA12814, NA12815, NA12891, NA12892), 12 Han Chinese samples (NA18524, NA18526, NA18537,NA18545, NA18552, NA18562, NA18566, NA18572, NA18577, NA18609, NA18621, NA18635), 11 Japanese (Tokyo) samples (NA18940, NA18942, NA18945, NA18949, NA18953, NA18961, NA18964, NA18967, NA18981, NA18994, NA18998), 22 samples of Hispanic origin (NA17438, NA17439, NA17440, NA17441, NA17442, NA17443, NA17444, NA17445, NA17446, NA17448, NA17449, NA17450, NA17451, NA17452, NA17453, NA17454, NA17456, NA17457, NA17458, NA17459, NA17460, NA17461, 15 samples of African American origin (NA17101, NA17102, NA17103, NA17104, NA17105, NA17106, NA17107, NA17108, NA17109, NA17110, NA17111, NA17112, NA17113, NA17114, NA17115) and 12 samples of Yoruban origin (NA18502, NA18504, NA18855, NA18870, NA19137, NA19144, NA19153, NA19200, NA19201, NA19203, NA19223, NA19238). A 200-bp amplicon was designed for each insertion. By design, a homozygous insertion sequence yielded a single high–molecular weight band of (200 bp + the insertion size) on the agarose gel. Absence of the insertion would be detected as a single low molecular band of 200 bp alone and a heterozygous indel would be detected as presence of both bands.

The amplicons were classified according to theoretical melting temperatures (*T*_m_). Standard GC content and high GC content amplicons (82 °C < *T*_m_ < 87 °C) were processed separately in the laboratory using optimized high-throughput PCR protocols enabling all amplifications to be performed in 384-well plates in a volume of 10 μl. The standard GC content PCR protocol was composed of 3.0 μl of 0.4 μM mixed forward and reverse primers, 3.0 μl of DNA (1.67 ng/μl) and 0.05 μl (0.25 Us) of AmpliTaq Gold DNA polymerase (Applied Biosystems). The high-GC PCR protocol comprised 3.0 μl of 1.2 μM mixed forward and reverse primers, 3.0 μl of DNA (10.0 ng/μl), and 0.075 μl (0.375 U) of AmpliTaq Gold DNA polymerase (Applied Biosystems). PCR was set up using a Biomek FX (Beckman Coulter) pipetting robot and a Pixsys 4200 (Cartesian Technologies; http://www.cartesiantech.com/) nanoliter dispenser. All PCR amplifications were performed on dual 384-well GeneAmp PCR System 9700 thermal cyclers (Applied Biosystems) under the following program: 96 °C for 5 min (1×); 94 °C for 30 s, 60 °C for 45 s, 72 °C for 45 s (40×); 72 °C for 10 min (1×); and a 10 °C final hold.

2.0 μl of PCR product was combined with 5.0 μl of diluted loading dye (Invitrogen) and run on a 2.0% agarose gel, containing ethidium bromide. Gels were run for 45 min at 90 V and imaged using a Gel Doc and Quantity One Software (Bio-Rad Laboratories). Gel images were manually evaluated for the presence or absence of expected products.

### Confirmation of large indels by mapping fosmid clones from multiple individuals.

Segments of the human genome that were found exclusively in either HuRef or NCBI version 36 represent potential misassemblies or genuine variations. In order to distinguish between these possibilities, we attempted to confirm the existence of the largest one-to-one HuRef–NCBI indels in a collection of fosmid clones, derived from eight individuals (see Table S5 legend). Fosmid end reads were downloaded from the Trace Archive, and mapped to HuRef and NCBI human reference genome using Snapper (http://sourceforge.net/projects/kmer/). To avoid short allelic variants of single loci, the HuRef assembly included only scaffolds that spanned at least 30 kb. The initial alignments required a unique best score with at least 90% nucleotide identity for at least 25% of the read length. Pairs of end read alignments were then filtered sequentially to retain only those that mapped to the same scaffold (HuRef genome) or chromosome (NCBI reference genome), in a tail-to-tail orientation, and within three standard deviations of the mean insert length. First, regions of the HuRef genome that failed to map to NCBI reference genome in the one-to-one mapping and were spanned to an average depth of 10x by fosmids that failed to map to the NCBI reference genome were identified as potentially novel segments. Their sequences were aligned to NCBI using ncbi-blastn (-W 100), and novelty was defined by the absence of nucleotide identity (≥98%) for lengths of ≥1 kb in spans of at least 35 kb. Second, the mapping coordinates of clones that mapped discordantly to either HuRef or NCBI were intersected with the 40 largest one-to-one HuRef-NCBI–derived indels to identify fosmid clones that support the existence of these indels in other human genomes. To define inserted DNA, we required one fosmid end to map within the insert exclusive to one assembly, the other to map within flanking sequence common to both assemblies, and inconsistent mapping to the genome assembly that lacked the insertion. Defining absence of inserted DNA required the fosmid mapping to span the putative insertion point in the assembly that lacked the insertion, and inconsistent mapping to the assembly that contains the insertion.

### Haplotype assembly.

Haplotypes of heterozygous variants were inferred using a greedy heuristic with iterative refinement of the initial solution.

*Data Encoding.* An SNP matrix (rows = reads or mate pairs, columns = variants) was constructed as follows: for each variant location, reads whose sequence matched the consensus sequence were assigned state “0,” while reads not matching the consensus were assigned state “1.” A pair of mated reads was merged into a single row only if they were in the same scaffold, with the expected orientation and separated by the expected distance (± 3 SD). Thus, a row in the matrix correspond to one of the following: (i) a pair of mated reads with consistent placements and (ii) a single unmated read or single mated read whose mate is not consistently placed.

*Initial Haplotype Construction.* Initial partial haplotypes were constructed by repeating the following sequence of steps until all rows were assigned. From the remaining set of unassigned rows (initially all), choose the row with fewest missing elements. Use this row to seed a partial haplotype pair (i.e., assign the row to one haplotype, which is initialized with the non-missing states from this row, and initialize the other haplotype with the complementary states). Until no more rows share non-missing information, identify the row that has the strongest signal (i.e., number of columns indicating one haplotype minus number of columns indicating the other haplotype is maximal), and assign that row to the indicated haplotype, extending the haplotypes to include any additional columns that are non-missing for that row. When no unassigned rows overlap the current haplotypes, consider this pair of partial haplotypes final and go back to the beginning.

*Iterative Haplotype Refinement.* When all rows have been assigned to partial haplotypes, each haplotype pair and the rows it includes can be refined iteratively, repeating the following two steps until no changes result. First, for each column (variant position) in the haplotypes, determine by majority rule the state assignment of each haplotype. Second, for each row (read or mate pair), determine the haplotype assignment by majority rule.

*Measurement of Haplotype Sizes.* For each pair of partial haplotypes, two measures of size are natural: the number of variants that are phased and the distance in bp from the first to the last variant. In addition to the average of such values, the N50 statistic indicates a haplotype size that encompasses at least half of the variants.

*Comparison of Phasing to HapMap.* Consistency of HuRef haplotypes with HapMap haplotypes was assessed as follows. Within each partial HuRef haplotype, variants that were present in Phase I HapMap data were identified (henceforth “HapMap variants”). For each pair of HapMap variants that were adjacent in a HuRef haplotype, two measures were determined. The first was the degree of LD between the paired variants from the HapMap CEU panel. The second was the conditional probability of observing the HuRef haplotype in the CEU panel given the observed genotypes. When *r* ^2^ ≥ 0.9 and the conditional probability was <0.5, this was considered a clear conflict of HuRef and HapMap haplotypes.

### Affymetrix microarray experiments and data analysis.

The HuRef sample was genotyped in duplicate on each of the GeneChip Human (500K) Mapping *Nsp*I and *Sty*I Array Sets (Affymetrix; http://www.affymetrix.com), according to the manufacturer's instructions and as described previously [96]. Each array contains an average of 250,000 SNP markers. The arrays were scanned using the Gene Chip Scanner 3000 7G and Gene Chip Operating System. The call rate was >96% for all four all hybridizations; 0.1% discordant genotype calls between the technical replicates were excluded from further analysis.

The *Nsp*I and *Sty*I array scans were analyzed for copy number variation using a combination of DNA Chip Analyzer (dChip) [97], Copy Number Analysis for GeneChip (CNAG) [98], and Genotyping Microarray-based CNV Analysis (GEMCA) [99]. Analysis with dChip (http://www.dchip.org) was performed using a Hidden Markov Model (HMM) as previously described [100], and a set of 50 samples run in the same facility were used as reference. For analyses with CNAG version 2.0 (http://www.genome.umin.jp), the copy number changes were inferred using a HMM built into CNAG [98]. GEMCA analysis was performed essentially as described [99], except that we used one designated DNA samples (NA10851) as reference for pair-wise comparison. This sample has been screened for CNVs in a previous study [62] and the CNVs known to be present in the reference genome were excluded.

### Illumina HumanHap650Y Genotyping BeadChip.

The HuRef sample was genotyped using the Sentrix HumanHap650Y Genotyping BeadChip according to the manufacturer's instructions. All chips were scanned using the Sentrix Bead-Array reader and the Sentrix Beadscan software application. The results from the BeadChip were analyzed for CNV content using QuantiSNP as previously described [101].

### Human genome Agilent 244K CGH arrays.

The Agilent human genome CGH array contains 244,000 60mer probes on a single slide. The experiment was run using 2.5 μg of genomic DNA for Cy3/Cy5 labeling for each hybridization, with a standard dye-swap experimental design. DNA sample NA10851 was used as a reference. The slides were scanned at 5-μm resolution using the Agilent G2565 Microarray Scanner System (Agilent Technologies; http://www.agilent.com). Feature extraction was performed using Feature Extraction v9.1 and results were analyzed using CGH Analytics v3.4.27.

### Nimblegen human whole-genome 385K CGH array.

CGH was performed using the Nimblegen human genome CGH array. The array contains 385,000 isothermal probes yielding a median spacing of 6 kb across the human genome. The experiment was performed as previously described [102] with a standard dye-swap experimental design. Results were analyzed using the CNVfinder algorithm [103]. One of the dye-swap experiments did not meet the quality control cut-offs, and because of this, the Nimblegen CNV calls were only employed for confirmation of CNV identified by the other platforms, and not used for identification of additional CNVs

### FISH.

FISH analysis was performed to find the location of DNA segments present in the HuRef DNA but either missing or represented by gaps in HuRef assembly. The FISH analysis was performed as previously described [104]. Initially, fosmids representing 107 different regions were chosen and end-sequenced to confirm that they mapped to the intended scaffolds. After excluding fosmids for which the original mapping was erroneous or uncertain, 88 fosmids remained. The entire sequence for each fosmid was then computationally excised from the scaffolds sequence and analyzed for repeat content using RepeatMasker. Fosmids with more than 6 kb (∼17%) satellite repeat content were excluded from further analysis. All fosmids that passed these filtering criteria were analyzed on metaphase spreads from two different cell lines (GM10851 and GM15510) to determine the chromosomal location of the fosmid probe. At least 10 metaphases were scored for each probe, all in duplicate by two experienced cytogeneticists.

## Supporting Information

Figure S1 Detection of an 8-bp Indel in the HuRef and Three Coriell DNA Donors PAGE detection of an 8-bp indel (GATAAGTG/--------) in three Coriell DNA samples (lane 1 = NA05392 , lane 2 = NA05398, lane 3 = NA07752, and lane 4 = HuRef donor DNA). Note the detection of two bands signifying the presence of two allelic forms in individual NA05392 and HuRef and the short and long alleles in individuals NA05398 and NA07752 respectively.
(118 KB PDF)

Figure S2 The Distribution of the Relative Position of SNPs and Heterozygous Indels Found in Sequence Reads Note the increased occurrence of variant at the beginning and end of reads. The relative position of increased rate of variant identification was used and reads calling variant outside these threshold were removed as positive evidence for the presence of that particular variant. This approach led to a significant improvement in the quality of the variant sets and was used as part of the variant filtering process.
(132 KB PDF)

Figure S3 The Distribution of the Percentage of Reads Supporting the Alternate (i.e., Non–Consensus Sequence Reads) for All Raw SNP and Heterozygous Indels The dbSNP distribution indicates which “raw” variant have been previously reported in dbSNP. The intersection point of these two distributions at lower values determines the minimum percentage of minor allele threshold with which variant could be filtered to improve their quality using dbSNP as a guide. These threshold values were deemed to be 25% for SNP and 20% for indels. Indels are referred to separately as insertions and deletions depending on whether the shorter or longer form, respectively, was used in the HuRef consensus sequence. Ultimately these variant loci are all determined heterozygous indels as indicated in Figure 4.
(121 KB PDF)

Table S1 Detailed Specification of Libraries Used for Sequencing of the HuRef Donor (82 KB DOC)

Table S2 Four-Way Comparison of the Genome Assemblies Using WGSA, HuRef all Scaffolds, HuRef Chromosomes, and Human NCBI Version 36 Matches are maximal high identity local aligned segments with no indels. Runs are sequentially adjacent matches with no intervening matches from other genomic sequences with the possibility of indels. Clumps are the same as runs but allow small intervening matches/runs to be skipped over in addition to indels (small is a settable parameter). This allows for example small inversions not to interrupt a longer alignment. All subsequent analyses (i.e., variant detection and analysis) discussed in the manuscript were performed using HuRef scaffolds. N50 is the scaffold length such that 50% of all base pairs are contained in scaffolds of this length or larger.
(64 KB DOC)

Table S3 Percentage Coverage of NCBI Chromosomes with HuRef Chromosomes Matches are maximal local identical alignments with no indels. Runs are monotonically increasing sets of matches with no intervening matches with indels allowed. The values in the table denote the percentage of the NCBI chromosome found in matches or runs counting alignments containing bases (i.e., no ambiguous or gaps, Ns.)
(44 KB DOC)

Table S4 AluY Insertions That Differ between the HuRef Genome and the NCBI Human Reference Genome (205 KB XLS)

Table S5 Mapping of Fosmid Clones from Multiple Individuals to the Sites of the Longest ATAC-Derived Indels Fosmid clones were mapped to the sites of large insertions that are predicted to occur uniquely on HuRef (insertions) or NCBI (deletions) as described in the Materials and Methods. For each example of inserted DNA, the table lists the assembly source (HuRef scaffold or NCBI chromosome), the start and end coordinates for the inserted DNA, the span of the inserted DNA, and the predicted insertion point on the assembly that lacks the insertion. Fosmid clones that support the presence or absence of inserted DNA are termed (+) insert fosmids and (−) insert fosmids, respectively. For each (+) insert fosmid, one end of the fosmid clone was mapped within the unique insert DNA while the other was mapped to common flanking DNA. For each (−) insert fosmid, the mapped ends span the site of insertion, but the implied insert length suggests that insert DNA is absent from the clone. Fosmid clones that support the presence (+) or absence (−) of inserted DNA were derived from the following Coriell cell lines; A, NA18517; B, NA18507; C, NA18956; D, NA19240, E, NA18555; F, NA12878; G, NA18552; H, NA15510.
(168 KB DOC)

Table S6 Homozygous Insertions Tested in 93 Coriell DNA Samples and the HuRef Donor DNA I/I provides the number of Coriell DNA samples that are homozygous for the inserted sequence, heterozygous (I/N), and homozygous for no insertion (N/N). The Hardy-Weinberg *p*-value is based on CEU individuals.
(95 KB DOC)

Table S7 CNV Identified in the Donor DNA Overlapping Genes (59 KB XLS)

Table S8 FISH Performed Using Coriell Fosmid Clones and Coriell DNA Metaphase Spreads (24 KB XLS)

Poster S1 The Diploid Genome Sequence of J. Craig Venter This genome-wide view attempts to illustrate the wide spectrum of DNA variation in the diploid chromosome set of an individual human, J. Craig Venter. The genome sequence is displayed on a nucleotide scale of approximately 1Mb/15 mm. The background of the chromosome tracks shows an approximate correspondence of features from the chromosome cytogenetic map. The different data tracks are grouped into two major sections: a representation of a current set of transcription units and a set of summary plots for different variation features at sequence level.
For each DNA strand (forward and reverse), each mapped gene is shown at genomic scale and is color-coded according to the presence of transcript isoforms (see Gene Variants panel on figure key). A total of 54,253 transcript isoforms were mapped. The genes are given a minimum length of 10 kb for display purposes at this level. The largest transcript isoform for all genes that are between 2.5 kb and 250 kb and have at least five exons are shown, in an additional pair of tracks, expanded to a resolution close to 100 kb /15 mm. Due to their high gene density, the resolution is smaller for Chromosomes 17, 19, and 22 at approximately 80 kb/15 mm.
In these expanded tiers, exons are depicted as black boxes and introns are color coded according to a set of Gene Ontology categories (GO, http://www.geneontology.org), as shown in the corresponding panel on the figure key. Gene symbols appear close to the corresponding expanded transcript when space permits, Ensembl transcript identifiers are shown if there is no Hugo gene symbol associated with that transcript. In order to produce more compact labels the “ENST0+” prefix has been replaced by “ET.” Between the forward and the reverse strand annotations, two color gradients show the nucleotide exon coverage detected in 5-kb-long sliding windows for each strand.
Below the reverse strand annotations track there is the copy number variation (CNV) track. Here, results from four different experimental platforms (Affymetrix, Illumina, Agilent, and Nimblegen) determine regions where a CNV gain or loss was detected, shown as green or red boxes respectively. Nonoverlapping haplotype blocks are distributed into nine tracks, using distinct colors to enhance visibility. The longest blocks at each given loci are drawn as yellow boxes with a red outline to highlight them from the rest. A summary of the variation features defined for all the haplotype blocks is shown as a gray-scale gradient. Alternating color gradient tracks display count densities for homozygous SNP, heterozygous SNP, multiple nucleotide (MNPs), insertion/deletion polymorphisms, and complex forms of variation. The last two gradients contain count densities for tandem repeats and transposon repeats respectively. All these color gradients were produced using a 5-kb sliding window.
The figure was generated with “gff2ps” (http://genome.imim.es/software/gfftools/GFF2PS.html), a genome annotation tool that converts General Feature Formatted records (http://www.sanger.ac.uk/Software/formats/ GFF/) to a PostScript output [J F Abril, R Guigó (2000) gff2ps: Visualizing genomic annotations. Bioinformatics 16: 743–744]. To navigate the interactive poster, go to http://journals.plos.org/plosbiology/suppinfo/pbio.0050254/sd001.php.
(88 MB PDF)

### Accession Numbers

The GenBank (http://www.ncbi.nlm.nih.gov/Genbank) accession number for sequences discussed in the paper are: AADD00000000 (WGSA) and ABBA01000000 (the consensus sequences of both HuRef scaffolds and chromosomes).

## Abbreviations

CGH: comparative genomic hybridization
CHB+JPT: grouped Han Chinese and Japanese
CNV: copy number variant
CEU: Caucasian
BAC: bacterial artificial chromosome
FISH: fluorescence in situ hybridization
LD: linkage disequilibrium
MNP: mulit-nucleotide polymorphism
QV: quality value
SINE: short interspersed nuclear element
SNP: single nucleotide polymorphism
WGSA: whole-genome shotgun assembly
YRI: Yoruban

## Note Added in Proof

References 105 and 106 were added at the proof stage and so are cited out of order in the text. During the review process we became aware of a recently published paper on haplotype assembly that deserves mention for its relatedness to our haplotype separation approaches [106].
