# Supplementary material for: The Diploid Genome Sequence of an Individual Human
Source: PLoS Biol. 2007 Sep 4;5(10):e254. doi: 10.1371/journal.pbio.0050254 (PMC1964779; doi:10.1371/journal.pbio.0050254)
Supplement: Supplementary file 1 [file pbio.0050254.sd001.pdf]

The Diploid Genome Sequence of J. Craig Venter

J. Craig Venter INSTITUTE www.jcvi.org

PLoS BIOLOGY www.plosbiology.org

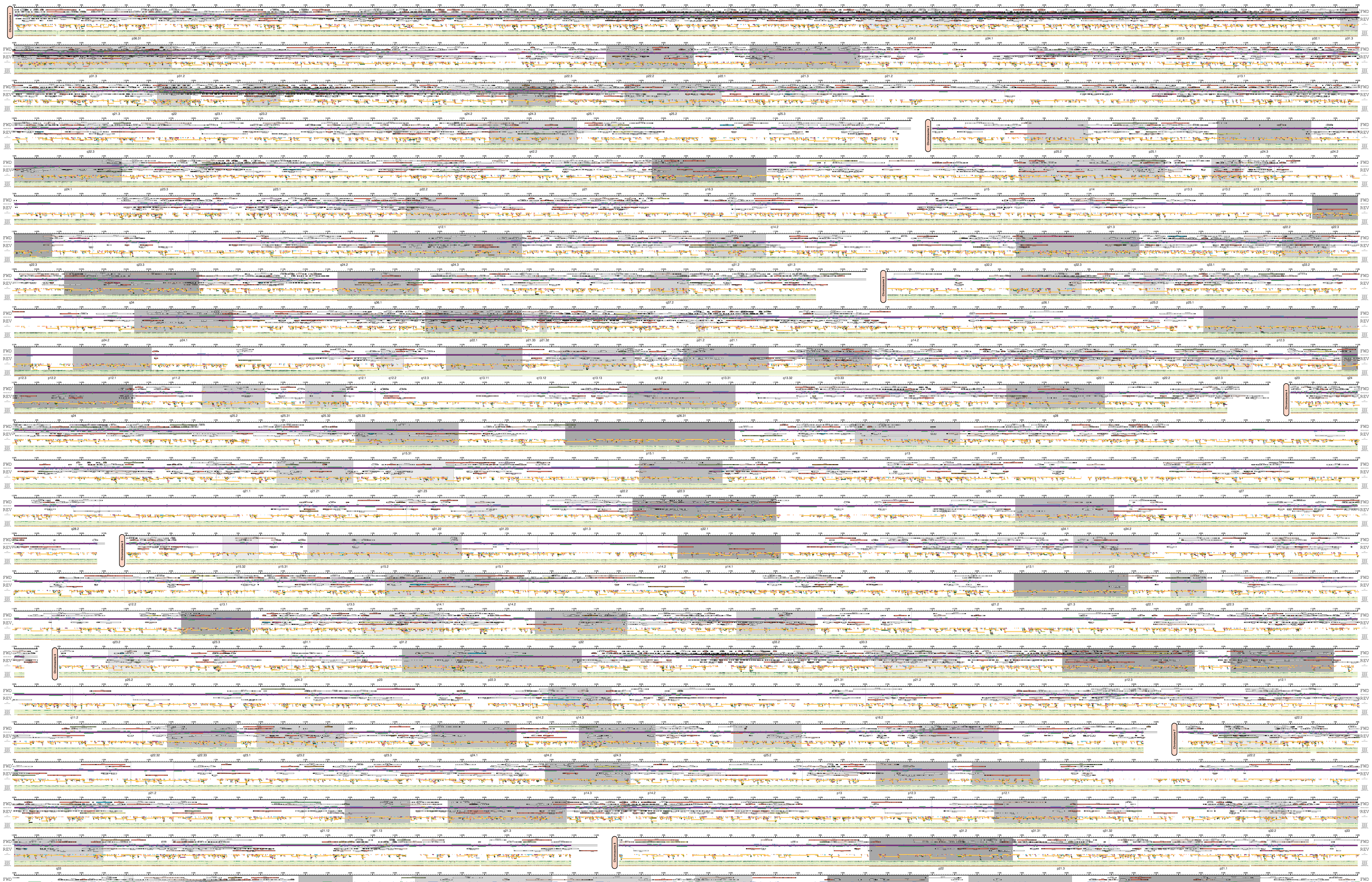

Poster S1. The Diploid Genome Sequence of J. Craig Venter

The Diploid Genome Sequence of an Individual Human

Levy, S. *et al* (2007) **PLoS Biology** 5(10):e254

This genome-wide view attempts to illustrate the wide spectrum of DNA variation in the diploid chromosome set of an individual human, J. Craig Venter. The genome sequence is displayed on a nucleotide scale of approximately 1 Mb/15mm. The background of the chromosome tracks shows an approximate correspondence of features from the chromosome cytogenetic map. The different data tracks are grouped into two major sections: a representation of a current set of transcription units and a set of summary plots for different variation features at sequence level.

For each DNA strand (forward and reverse), each mapped gene is shown at genomic scale and is color-coded according to the presence of transcript isoforms (see GENE VARIANTS panel on figure key). A total of 54,253 transcript isoforms were mapped. The genes are given a minimum length of 10 kb for display purposes at this level. The largest transcript isoform for all genes that are between 2.5 kb and 250 kb, and have at least five exons, are shown in an additional pair of tracks, expanded to a resolution close to 100 kb /15mm. Due to their high gene density, the resolution is smaller for chromosomes 17, 19 and 22 at approximately 80 kb/15mm).

In these expanded tiers, exons are depicted as black boxes and introns are color coded according to a set of Gene Ontology categories (GO, http://www.geneontology.org), as shown in the corresponding panel on the figure key. Gene symbols appear close to the corresponding expanded transcript when space permits, ENSEMBL transcript identifiers are shown if there is no HUGO gene symbol associated with that transcript. In order to produce more compact labels the "ENST0+" prefix has been replaced by "ET". Between the forward and the reverse strand annotations, two color gradients show the nucleotide exon coverage detected in 5 kb long sliding windows for each strand.

Below the reverse strand annotations track there is the Copy Number Variation (CNV) track. Here, results from four different experimental platforms (Affymetrix, Illumina, Agilent and Nimblegen) determine regions where a CNV gain or loss was detected, shown as green or red boxes respectively. Non-overlapping haplotype blocks are distributed into nine tracks, using distinct colors in order to enhance visibility. The longest blocks at each given loci are drawn as yellow boxes with a red outline to highlight them from the rest. A summary of the variation features defined for all the haplotype blocks is shown as a grey scale gradient.

Alternating color gradient tracks display count densities for homozygous SNP, heterozygous SNP, multiple nucleotide (MNPs), insertion/deletion polymorphisms and complex forms of variation. The last two gradients contain count densities for tandem repeats and transposon repeats respectively. All these color gradients were produced using a 5 kb sliding window. The figure was generated with "gff2ps" (http://genome.imim.es/software/gfftools/GFF2PS.html), a genome annotation tool that converts General Feature Formatted records (http://www.sanger.ac.uk/Software/formats/GFF/) to a PostScript output [J. F. Abril, R. Guigó, **Bioinformatics** 16, 743 (2000)].

SOME RIGHTS RESERVED

PLoS Biology is the world's premier peer-reviewed, open-access journal published by the Public Library of Science.

OPEN ACCESS **Freely available online**

Download this poster and its corresponding research article at www.plosbiology.org

Poster Credits: Josep F. Abril, Samuel Levy, Brian P. Walenz, Pauline C. Ng, Nelson Axelrod, Aaron L. Halpern, Roderic Guigó, Marvin E Frasier, Robert L. Strausberg and J. Craig Venter

DOI: 10.1371/journal.pbio.0050254
