# Supplementary material for: The Diploid Genome Sequence of an Individual Human
Source: PLoS Biol. 2007 Sep 4;5(10):e254. doi: 10.1371/journal.pbio.0050254 (PMC1964779; doi:10.1371/journal.pbio.0050254)
Supplement: Supplementary file 3 [file pbio.0050254.sg002.pdf]

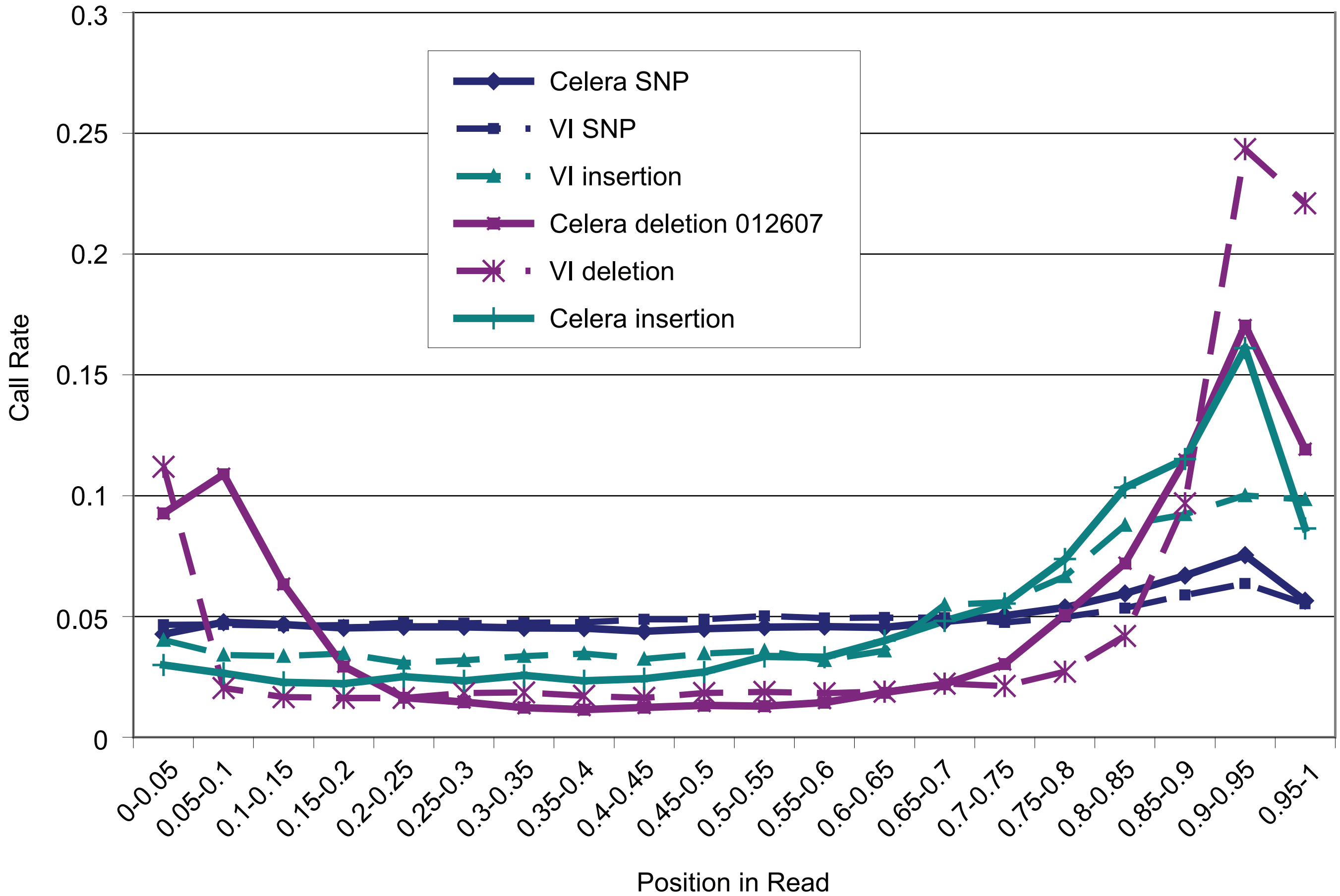

Call Rate
0.3
0.25
0.2
0.15
0.1
0.05
0
Celera SNP
VI SNP
VI insertion
Celera deletion 012607
VI deletion
Celera insertion
0-0.05
0.05-0.1
0.1-0.15
0.15-0.2
0.2-0.25
0.25-0.3
0.3-0.35
0.35-0.4
0.4-0.45
0.45-0.5
0.5-0.55
0.55-0.6
0.6-0.65
0.65-0.7
0.7-0.75
0.75-0.8
0.8-0.85
0.85-0.9
0.9-0.95
0.95-1
Position in Read

Figure S2
