# Supplementary material for: The Diploid Genome Sequence of an Individual Human
Source: PLoS Biol. 2007 Sep 4;5(10):e254. doi: 10.1371/journal.pbio.0050254 (PMC1964779; doi:10.1371/journal.pbio.0050254)
Supplement: Supplementary file 4 [file pbio.0050254.sg003.pdf]

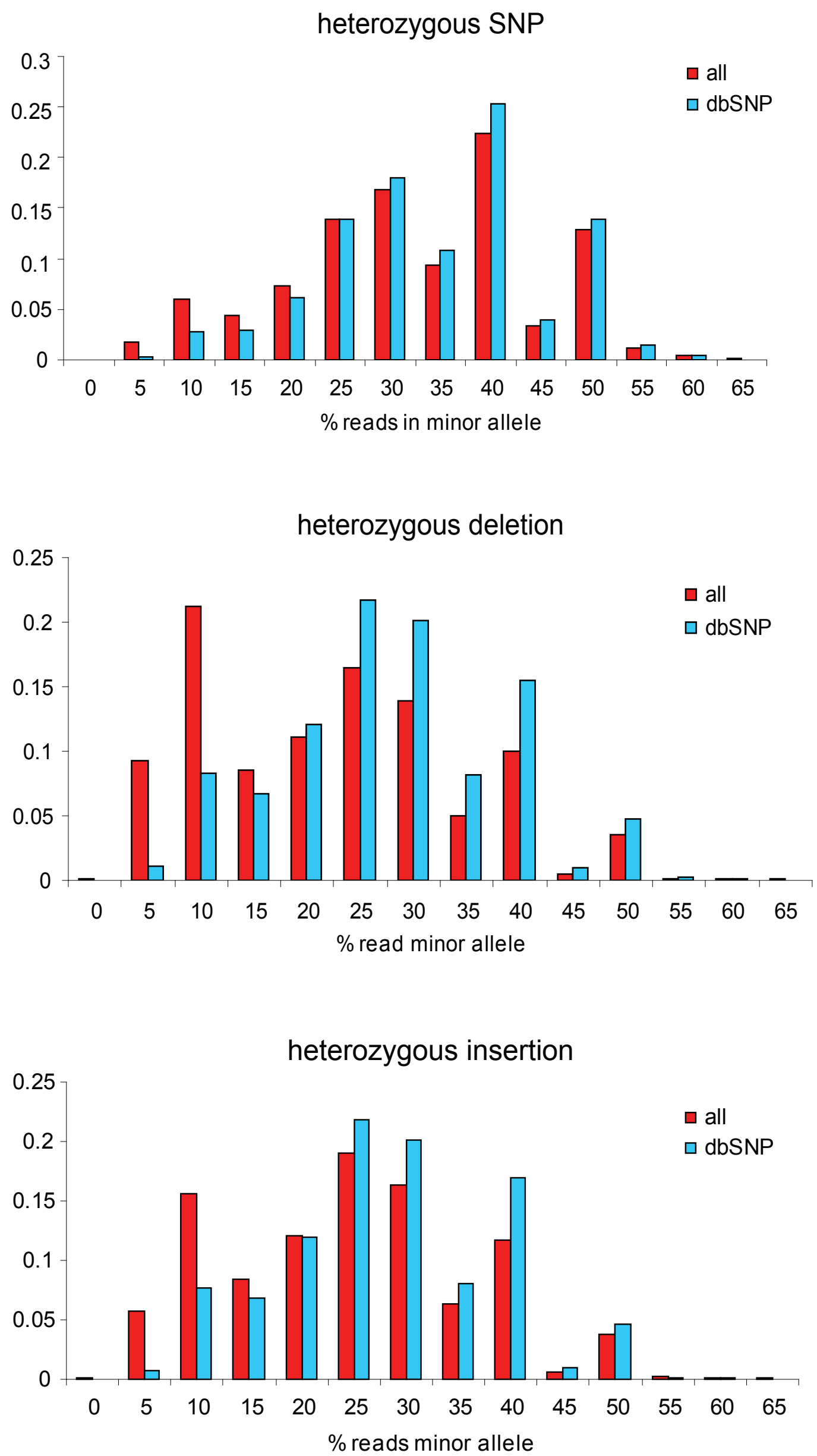
heterozygous SNP
0.3
0.25
0.2
0.15
0.1
0.05
0
all
dbSNP
0 5 10 15 20 25 30 35 40 45 50 55 60 65
% reads in minor allele
heterozygous deletion
0.25
0.2
0.15
0.1
0.05
0
all
dbSNP
0 5 10 15 20 25 30 35 40 45 50 55 60 65
% read minor allele
heterozygous insertion
0.25
0.2
0.15
0.1
0.05
0
all
dbSNP
0 5 10 15 20 25 30 35 40 45 50 55 60 65
% reads minor allele

Figure S3
