# Supplementary material for: The Diploid Genome Sequence of an Individual Human
Source: PLoS Biol. 2007 Sep 4;5(10):e254. doi: 10.1371/journal.pbio.0050254 (PMC1964779; doi:10.1371/journal.pbio.0050254)
Supplement: Supplementary file 5 [file pbio.0050254.st001.doc]

| **Library type** | **Mean** | **Std.Dev.** | **Number Reads** | **Number Clones** | **Read Coverage** | **Clone Coverage** |
| --- | --- | --- | --- | --- | --- | --- |
| 01-Huref-12KB | 16,095.42 | 1,783.99 | 657,975 | 328,128 | 0.182 | 1.428 |
| 02-Huref-13KB | 19,282.08 | 2,742.41 | 768 | 384 | 0.000 | 0.002 |
| 03-Huref-10-12kb | 9,672.07 | 998.26 | 1,326,230 | 661,344 | 0.359 | 1.730 |
| 04-Huref-10-12kb | 11,394.32 | 2,673.83 | 768 | 384 | 0.000 | 0.001 |
| HUREF-B-03-120KB | 91,451.72 | 22,760.15 | 390,101 | 194,655 | 0.112 | 4.813 |
| HuRef-F-0140kb | 37,506.11 | 2,983.56 | 768 | 375 | 0.000 | 0.004 |
| HuRef-F-02-40kb | 37,323.66 | 3,926.69 | 1,535 | 767 | 0.000 | 0.008 |
| HuRef-F-04-40Kb | 36,476.82 | 3,677.18 | 98,092 | 48,940 | 0.027 | 0.483 |
| HuRef-F-06-40kb | 36,264.40 | 3,244.97 | 419,231 | 208,808 | 0.113 | 2.048 |
| HuRef-F-07-40kb | 35,581.33 | 2,952.15 | 612,868 | 305,130 | 0.162 | 2.936 |
| HuRef-F-08-40kb | 39,459.32 | 3,313.57 | 709,561 | 353,239 | 0.186 | 3.769 |
| HuRef-F-09-40kb | 36,579.63 | 3,692.00 | 86,725 | 43,337 | 0.023 | 0.429 |
| HuRef-F-10-40kb | 36,835.83 | 3,584.54 | 426,477 | 212,222 | 0.116 | 2.114 |
| HuRef-F-11-40kb | 36,427.13 | 3,590.94 | 126,895 | 63,152 | 0.034 | 0.622 |
| HuRef-F-12-40kb | 37,346.61 | 3,641.69 | 130,256 | 65,018 | 0.035 | 0.657 |
| HuRef-F-13-40kb | 35,517.74 | 3,037.59 | 67,401 | 33,687 | 0.018 | 0.324 |
| HuRef-F-14-40kb | 38,557.74 | 3,655.59 | 170,124 | 84,879 | 0.046 | 0.885 |
| HuRef-F-15-40kb | 38,209.23 | 3,466.00 | 3,840 | 1,920 | 0.001 | 0.020 |
| HuRef-F-17-40kb | 37,265.88 | 3,734.52 | 3,826 | 1,906 | 0.001 | 0.019 |
| HuRef-F-McBrc-40Kb | 38,392.00 | 4,028.31 | 768 | 384 | 0.000 | 0.004 |
| HuRef-F-McBrc2-40kb | 38,360.31 | 4,101.47 | 767 | 383 | 0.000 | 0.004 |
| Huref-F-16-40kb | 36,640.55 | 3,621.71 | 3,801 | 1,882 | 0.001 | 0.019 |
| Huref-F-20-40kb | 37,452.61 | 3,504.09 | 3,838 | 1,918 | 0.001 | 0.019 |
| Huref-F-21-40kb | 37,293.48 | 3,434.86 | 2,304 | 1,152 | 0.001 | 0.012 |
| Huref-F-22-40kb | 36,354.73 | 3,625.52 | 3,836 | 1,917 | 0.001 | 0.019 |
| T14116-JA-8P5-10P5KB | 12,025.12 | 1,190.24 | 1,595,726 | 790,319 | 0.414 | 2.570 |
| T14117-JB-11-14KB | 9,290.53 | 692.72 | 3,272,048 | 1,612,934 | 0.844 | 4.052 |
| T14426-JC-9-11KB | 11,659.08 | 1,289.79 | 2,491,702 | 1,214,872 | 0.674 | 3.830 |
| T14427-JD-10-12KB | 13,460.73 | 1,563.09 | 768 | 384 | 0.000 | 0.001 |
| CRA-HUBB101S-10201 | 10,242.12 | 756.30 | 7,515,891 | 1,901,255 | 1.637 | 5.265 |
| CRA-HUBB2KB-1925 | 1,950.51 | 98.47 | 11,737,820 | 3,413,119 | 2.563 | 1.800 |
