# Supplementary material for: The Diploid Genome Sequence of an Individual Human
Source: PLoS Biol. 2007 Sep 4;5(10):e254. doi: 10.1371/journal.pbio.0050254 (PMC1964779; doi:10.1371/journal.pbio.0050254)
Supplement: Supplementary file 6 [file pbio.0050254.st002.doc]

| **A** | NCBI - All | NCBI - chr | NCBI - chr | WGSA - chr |
| --- | --- | --- | --- | --- |
| **B** | HuRef - All | WGSA - chr | HuRef - chr | HuRef - chr |
|  |  |  |  |  |
| **matches** |  |  |  |  |
| Number | 802,349 | 1,207,288 | 794,236 | 907,701 |
| Number bp | 2,768,534,560 | 2,640,396,081 | 2,759,247,934 | 2,639,181,916 |
| Number ACGT bp A | 2,768,534,394 | 2,640,396,058 | 2,759,247,906 | 2,639,181,916 |
| Number ACGT bp B | 2,768,534,520 | 2,640,396,041 | 2,759,247,894 | 2,689,181,916 |
|  |  |  |  |  |
| N50 | 6,337 | 4,121 | 6,379 | 6,280 |
|  |  |  |  |  |
| **runs** |  |  |  |  |
| Number | 25,845 | 7,724 | 4,470 | 8,073 |
| Number bp A | 2,789,788,352 | 2,752,986,879 | 2,800,937,689 | 2,752,429,194 |
| Number ACGT bp A | 2,789,187,467 | 2,750,031,631 | 2,797,592,941 | 2,654,626,229 |
| Number bp B | 2,791,629,568 | 2,746,311,764 | 2,792,161,644 | 2,757,563,751 |
| Number ACGT bp B | 2,776,267,848 | 2,648,326,131 | 2,768,718,662 | 2,737,522,540 |
| N50 | 722,849 | 1,769,135 | 6,379,829 | 1,702,556 |
|  |  |  |  |  |
| **clumps 5k** |  |  |  |  |
| Number | 15,191 | 480 | 350 | 394 |
| Number bp A | 2,803,278,106 | 2,771,626,012 | 2,828,706,701 | 2,774,528,406 |
| Number ACGT bp A | 2,801,940,806 | 2,766,361,264 | 2,821,092,453 | 2,656,617,856 |
| Number bp B | 2,812,317,827 | 2,770,880,410 | 2,802,225,543 | 2,774,540,298 |
| Number ACGT bp B | 2,785,816,586 | 2,653,167,307 | 2,775,567,329 | 2,751,357,628 |
|  |  |  |  |  |
| N50 | 12,428,550 12,428,550 | 29,404,101 29,404,101 | 47,774,195 47,774,195 | 28,945,369 28,945,369 |
|  |  |  |  |  |
| **clumps 50k** |  |  |  |  |
| Number | 14,987 | 249 | 121 | 228 |
| Number bp A | 2,803,107,553 | 2,770,956,757 | 2,828,882,176 | 2,775,100,152 |
| Number ACGT bp A | 2,801,681,396 | 2,765,692,009 | 2,820,967,928 | 2,655,231,639 |
| Number bp B | 2,812,130,146 | 2,769,161,835 | 2,802,206,043 | 2,772,386,130 |
| Number ACGT bp B | 2,785,616,240 | 2,651,549,779 | 2,775,451,643 | 2,749,335,957 |
| N50 | 13,024,261 | 33,346,136 | 57,402,262 | 36,595,718 |
