# Supplementary material for: The Diploid Genome Sequence of an Individual Human
Source: PLoS Biol. 2007 Sep 4;5(10):e254. doi: 10.1371/journal.pbio.0050254 (PMC1964779; doi:10.1371/journal.pbio.0050254)
Supplement: Supplementary file 7 [file pbio.0050254.st003.doc]

| Chr | Matches | Runs |
| --- | --- | --- |
| 1 | 96.26 | 97.51 |
| 2 | 97.61 | 98.77 |
| 3 | 99.30 | 99.88 |
| 4 | 98.79 | 99.47 |
| 5 | 97.86 | 98.68 |
| 6 | 98.90 | 99.62 |
| 7 | 96.60 | 98.02 |
| 8 | 98.25 | 99.01 |
| 9 | 90.35 | 91.71 |
| 10 | 96.60 | 97.59 |
| 11 | 98.79 | 99.49 |
| 12 | 98.99 | 99.76 |
| 13 | 98.98 | 99.66 |
| 14 | 98.12 | 98.87 |
| 15 | 94.94 | 96.88 |
| 16 | 93.54 | 95.74 |
| 17 | 96.00 | 98.00 |
| 18 | 98.99 | 99.64 |
| 19 | 96.97 | 99.32 |
| 20 | 98.89 | 99.96 |
| 21 | 96.96 | 98.22 |
| 22 | 94.47 | 96.29 |
| X | 89.37 | 95.21 |
| Y | 54.82 | 58.98 |
