# Supplementary material for: The Diploid Genome Sequence of an Individual Human
Source: PLoS Biol. 2007 Sep 4;5(10):e254. doi: 10.1371/journal.pbio.0050254 (PMC1964779; doi:10.1371/journal.pbio.0050254)
Supplement: Supplementary file 8 [file pbio.0050254.st005.doc]

| **HuRef Insertion (Unique Identifier)** | (+) insert Huref scaffold | | (+) insert start | (+) insert end | (+) insert span | (-) insert NCBI chromosome | (-) insert coordinate | (+) insert fosmids | (-) insert fosmids | A +/- | B +/- | C +/- | D +/- | E +/- | F +/- | G +/- | H +/- |
| --- | --- | --- | --- | --- | --- | --- | --- | --- | --- | --- | --- | --- | --- | --- | --- | --- | --- |
| 1104685392412 | 1103279188263 | 1,428,889 | | 1,511,600 | 82,711 | chr6 | 61,972,470 | 92 | 0 | 16/0 | 17/0 | 18/0 | 7/0 | 6/0 | 8/0 | 8/0 | 12/0 |
| 1104685230812 | 1103279188145 | 38,324,287 | | 38,389,311 | 65,024 | chr6 | 119,258,178 | 165 | 0 | 18/0 | 40/0 | 13/0 | 21/0 | 10/0 | 29/0 | 22/0 | 12/0 |
| 1104685619391 | 1103279188392 | 797,129 | | 859,502 | 62,373 | chr11 | 49,995,938 | 152 | 0 | 18/0 | 26/0 | 24/0 | 23/0 | 16/0 | 19/0 | 13/0 | 13/0 |
| 1104685525271 | 1103279188360 | 17,190,309 | | 17,231,496 | 41,187 | chr20 | 53,556,643 | 133 | 0 | 20/0 | 36/0 | 13/0 | 21/0 | 11/0 | 14/0 | 11/0 | 7/0 |
| 1104685338702 | 1103279188232 | 3,474,226 | | 3,499,814 | 25,588 | chr10 | 27,645,775 | 61 | 12 | 6/0 | 16/0 | 10/0 | 12/0 | 2/6 | 8/3 | 0/3 | 7/0 |
| 1104685552590 | 1103279188372 | 1,947,384 | | 1,971,660 | 24,276 | chr22 | 22,182,619 | 86 | 34 | 12/0 | 11/5 | 0/19 | 10/6 | 13/0 | 16/0 | 17/0 | 7/4 |
| 1104685034164 | 1103279181862 | 19,914,108 | | 19,931,088 | 16,980 | chr2 | 169,432,778 | 53 | 14 | 7/0 | 8/9 | 9/0 | 14/0 | 4/0 | 3/3 | 3/2 | 5/0 |
| 1104685567483 | 1103279188377 | 5,902,936 | | 5,919,377 | 16,441 | chr7 | 12,166,441 | 3 | 60 | 3/8 | 0/13 | 0/12 | 0/6 | 0/5 | 0/6 | 0/4 | 0/6 |
| 1104685338701 | 1103279188232 | 3,459,305 | | 3,474,197 | 14,892 | chr10 | 27,645,804 | 36 | 14 | 4/0 | 12/0 | 5/0 | 8/0 | 4/6 | 2/5 | 0/3 | 1/0 |
| 1104685575020 | 1103279188379 | 611,594 | | 624,194 | 12,600 | chr21 | 14,733,687 | 44 | 0 | 3/0 | 13/0 | 7/0 | 10/0 | 3/0 | 6/0 | 1/0 | 1/0 |
| 1104685383292 | 1103279188258 | 7,959,650 | | 7,971,791 | 12,141 | chr15 | 64,181,653 | 88 | 0 | 5/0 | 14/0 | 5/0 | 12/0 | 10/0 | 15/0 | 15/0 | 12/0 |
| 1104685552277 | 1103279188372 | 964,072 | | 976,005 | 11,933 | chr22 | 21,208,336 | 38 | 0 | 2/0 | 5/0 | 1/0 | 14/0 | 4/0 | 1/0 | 5/0 | 6/0 |
| 1104685256488 | 1103279188157 | 7,462,247 | | 7,473,694 | 11,447 | chr1 | 196,742,448 | 29 | 2 | 4/0 | 4/0 | 1/1 | 2/0 | 3/0 | 6/1 | 8/0 | 1/0 |
| 1104685103434 | 1103279187577 | 326,296 | | 337,426 | 11,130 | chr9 | 90,004,736 | 26 | 21 | 3/0 | 9/0 | 0/7 | 2/6 | 6/0 | 3/0 | 1/3 | 2/5 |
| 1104685161167 | 1103279188119 | 2,191,871 | | 2,202,940 | 11,069 | chr11 | 56,227,154 | 30 | 1 | 3/0 | 5/1 | 8/0 | 3/0 | 1/0 | 1/0 | 4/0 | 5/0 |
| 1104685273476 | 1103279188167 | 3,461,625 | | 3,471,753 | 10,128 | chr11 | 7,792,161 | 21 | 12 | 2/3 | 7/5 | 3/1 | 2/1 | 0/2 | 3/0 | 1/0 | 3/0 |
| 1104685408056 | 1103279188274 | 12,426,275 | | 12,436,352 | 10,077 | chr6 | 83,026,393 | 31 | 0 | 5/0 | 11/0 | 4/0 | 4/0 | 1/0 | 0/0 | 3/0 | 3/0 |
| 1104685581010 | 1103279188379 | 19,744,943 | | 19,754,478 | 9,535 | chr21 | 33,768,967 | 42 | 0 | 3/0 | 5/0 | 3/0 | 6/0 | 7/0 | 6/0 | 7/0 | 5/0 |
| 1104685294718 | 1103279188187 | 9,200,111 | | 9,209,567 | 9,456 | chr3 | 75,546,048 | 49 | 0 | 3/0 | 5/0 | 8/0 | 12/0 | 4/0 | 2/0 | 12/0 | 3/0 |
| 1104685242016 | 1103279188147 | 18,051,629 | | 18,060,473 | 8,844 | chr12 | 56,755,766 | 23 | 0 | 1/0 | 9/0 | 4/0 | 3/0 | 0/0 | 3/0 | 2/0 | 1/0 |

Supplementary Table 5

| **HuRef Deletion (Unique Identifier)** | (+) insert NCBI chromosome | (+) insert start | (+) insert end | (+) insert span | (-) insert Huref scaffold | (-) insert coordinate | (+) insert fosmids | (-) insert fosmids | A +/- | B +/- | C +/- | D +/- | E +/- | F +/- | G +/- | H +/- |
| --- | --- | --- | --- | --- | --- | --- | --- | --- | --- | --- | --- | --- | --- | --- | --- | --- |
| 1104685360405 | chr13 | 56,625,006 | 56,644,717 | 19,711 | 1103279188250 | 2,820,248 | 0 | 6 | 0/0 | 0/6 | 0/0 | 0/0 | 0/0 | 0/0 | 0/0 | 0/0 |
| 1104685478737 | chr1 | 205,785,706 | 205,804,262 | 18,556 | 1103279188323 | 15,516,712 | 3 | 41 | 1/0 | 0/2 | 0/4 | 0/13 | 0/7 | 0/0 | 0/7 | 2/8 |
| 1104685125828 | chr2 | 19,058,768 | 19,077,252 | 18,484 | 1103279187996 | 1,938,964 | 0 | 95 | 0/10 | 0/23 | 0/11 | 0/10 | 0/8 | 0/12 | 0/9 | 0/12 |
| 1104685330381 | chr9 | 72,507,800 | 72,525,781 | 17,981 | 1103279188227 | 2,329,693 | 0 | 84 | 0/20 | 0/16 | 0/10 | 0/11 | 0/6 | 0/10 | 0/7 | 0/4 |
| 1104685558389 | chrX | 134,780,239 | 134,797,489 | 17,250 | 1103279188374 | 6,801 | 2 | 11 | 0/5 | 0/2 | 0/0 | 0/0 | 0/2 | 0/0 | 0/2 | 2/0 |
| 1104685360406 | chr13 | 56,612,326 | 56,625,000 | 12,674 | 1103279188250 | 2,820,254 | 4 | 6 | 0/0 | 0/6 | 1/0 | 0/0 | 0/0 | 0/0 | 2/0 | 1/0 |
| 1104685056026 | chr4 | 48,823,220 | 48,835,759 | 12,539 | 1103279184029 | 3,062 | 20 | 0 | 1/0 | 13/0 | 0/0 | 3/0 | 0/0 | 0/0 | 3/0 | 0/0 |
| 1104685659416 | chr10 | 124,350,230 | 124,362,406 | 12,176 | 1103279188397 | 640,960 | 11 | 45 | 1/13 | 1/6 | 0/2 | 6/0 | 0/2 | 3/1 | 0/9 | 0/12 |
| 1104685093410 | chr5 | 49,456,187 | 49,467,597 | 11,410 | 1103279187428 | 2,635 | 3 | 0 | 0/0 | 1/0 | 2/0 | 0/0 | 0/0 | 0/0 | 0/0 | 0/0 |
| 1104685194813 | chr8 | 32,799,551 | 32,810,807 | 11,256 | 1103279188135 | 6,085,630 | 2 | 52 | 0/3 | 2/5 | 0/9 | 0/5 | 0/12 | 0/7 | 0/6 | 0/5 |
| 1104685095462 | chr5 | 57,359,235 | 57,369,537 | 10,302 | 1103279187428 | 7,903,953 | 3 | 61 | 0/5 | 0/12 | 0/3 | 3/3 | 0/2 | 0/18 | 0/8 | 0/10 |
| 1104685594247 | chr3 | 194,358,026 | 194,368,102 | 10,076 | 1103279188385 | 1,156,448 | 31 | 39 | 3/2 | 10/5 | 3/0 | 3/6 | 5/2 | 7/8 | 0/8 | 0/8 |
| 1104685066002 | chr7 | 100,425,739 | 100,434,238 | 8,499 | 1103279185247 | 1,463,380 | 33 | 22 | 0/1 | 12/3 | 4/2 | 3/1 | 2/6 | 2/2 | 3/7 | 7/0 |
| 1104685399967 | chr11 | 101,071,326 | 101,079,824 | 8,498 | 1103279188268 | 19,186,981 | 4 | 44 | 2/4 | 2/1 | 0/10 | 0/6 | 0/7 | 0/7 | 0/8 | 0/1 |
| 1104685638772 | chr3 | 114,226,773 | 114,234,972 | 8,199 | 1103279188393 | 28,530,913 | 32 | 0 | 0/0 | 2/0 | 10/0 | 3/0 | 6/0 | 5/0 | 4/0 | 2/0 |
| 1104685730507 | chr4 | 147,139,031 | 147,147,193 | 8,162 | 1103279188415 | 7,130,948 | 29 | 2 | 1/2 | 5/0 | 6/0 | 2/0 | 5/0 | 3/0 | 3/0 | 4/0 |
| 1104685417429 | chr17 | 49,207,793 | 49,215,746 | 7,953 | 1103279188280 | 7,096,929 | 19 | 5 | 5/0 | 5/0 | 3/0 | 2/0 | 0/0 | 0/5 | 2/0 | 2/0 |
| 1104685097434 | chr7 | 154,022,834 | 154,030,686 | 7,852 | 1103279187493 | 707,694 | 9 | 27 | 3/0 | 4/1 | 0/1 | 0/4 | 0/5 | 1/0 | 0/11 | 1/5 |
| 1104685106643 | chr19 | 8,712,861 | 8,720,323 | 7,462 | 1103279187645 | 4,361 | 3 | 6 | 0/0 | 0/2 | 2/0 | 0/2 | 0/1 | 1/1 | 0/0 | 0/0 |
| 1104685646164 | chr5 | 83,984,081 | 83,991,043 | 6,962 | 1103279188395 | 13,237,468 | 7 | 24 | 3/0 | 3/1 | 0/2 | 1/0 | 0/5 | 0/8 | 0/6 | 0/2 |
