# Supplementary material for: The Diploid Genome Sequence of an Individual Human
Source: PLoS Biol. 2007 Sep 4;5(10):e254. doi: 10.1371/journal.pbio.0050254 (PMC1964779; doi:10.1371/journal.pbio.0050254)
Supplement: Supplementary file 9 [file pbio.0050254.st006.doc]

| **NCBI 36 chr** | **NCBI 36 begin** | **Length** | **Gene name and location in gene model** | **I/I** | **I/N** | **N/N** | **Huref Geno-type** | **Hardy-Weinberg**  **(p-value)^1^** |
| --- | --- | --- | --- | --- | --- | --- | --- | --- |
| 6 | 116,475,460 | 549 | FRK  INTRON.1 | 93 | 0 | 0 | I/I | 1.00 |
| 2 | 210,529,278 | 213 | Q96JI4_HUMAN  INTRON.33 | 93 | 0 | 0 | I/I | 1.00 |
| 9 | 95,010,855 | 477 | WNK2  INTRON.1 | 93 | 0 | 0 | I/I | 1.00 |
| 18 | 59,083,121 | 104 | BCL2  INTRON.2 | 93 | 0 | 0 | I/I | 1.00 |
| 4 | 164,977,211 | 224 | MARCH1  INTRON.1 | 89 | 2 | 2 | I/I | 1.00 |
| 3 | 141,157,144 | 206 | CLSTN2  INTRON.1 | 80 | 2 | 4 | I/I | 1.00 |
| 2 | 63,632,740 | 327 | NP_056994  INTRON.1 | 78 | 2 | 13 | I/I | 0.00 |
| 3 | 43,540,785 | 146 | TMEM16K  INTRON.10 | 73 | 16 | 4 | I/I | 1.00 |
| 4 | 44,114,389 | 1030 | KCTD8  INTRON.1 | 63 | 1 | 29 | I/I | 0.00 |
| 19 | 20,537,828 | 317 | BC015765  INTRON.1 | 60 | 24 | 9 | I/I | 0.74 |
| 10 | 60,654,927 | 331 | PHYHIPL  INTRON.1 | 52 | 30 | 11 | I/I | 0.36 |
| 15 | 98,628,516 | 309 | ADAMTS17  INTRON.3 | 47 | 7 | 39 | I/I | 0.00 |
| 11 | 123,626,497 | 851 | OR8G5  INTRON.1 | 29 | 0 | 64 | I/I | 0.00 |
| 7 | 138,255,459 | 255 | KIAA1549  UPSTREAM 3.4 kb | 29 | 6 | 57 | I/I | 0.16 |
| 12 | 40,133,990 | 241 | PDZRN4  INTRON.1 | 28 | 40 | 24 | I/I | 0.31 |
| 4 | 169,896,399 | 447 | PALLD  INTRON.10 | 25 | 37 | 31 | I/I | 0.27 |
| 6 | 96,615,901 | 324 | FUT9  INTRON.1 | 22 | 48 | 22 | I/I | 0.46 |
| 4 | 72,863,553 | 327 | CDH23  INTRON.1 | 20 | 37 | 33 | I/I | 1.00 |
| 12 | 129,710,823 | 470 | RIMBP2  INTRON.1 | 18 | 56 | 19 | I/I | 0.19 |
| 4 | 37,579,706 | 135 | TBC1D1  INTRON.1 | 13 | 45 | 35 | I/I | 0.38 |
| 7 | 48,491,725 | 489 | ABCA13  INTRON.45 | 12 | 25 | 56 | I/I | 0.76 |
| 4 | 36,001,600 | 320 | Q6ZMT9_HUMAN  INTRON.7 | 0 | 17 | 76 | I/I | 0.45 |
| 9 | 74,640,439 | 288 | TMC1  INTRON.21 | 1 | 4 | 88 | I/N | 0.82 |
| 19 | 58,001,661 | 317 | ZNF28  INTRON.3 | 2 | 17 | 74 | I/N | 0.91 |
| 12 | 26,849,927 | 399 | ITPR2  INTRON.1 | 33 | 22 | 38 | I/N | 0.13 |
| 15 | 82,744,230 | 197 | NP_443151  3'UTR | 74 | 14 | 1 | I/N | 0.82 |
| 15 | 98,157,482 | 309 | C15orf51  3'UTR | 3 | 20 | 50 | I/N | 1.00 |
| 20 | 45,728,510 | 172 | SULF2  EXON.12 | 0 | 33 | 60 | I/N | 0.04 |
| 5 | 16,769,627 | 310 | MYO10  INTRON.19 | 20 | 32 | 41 | I/N | 0.17 |
| 1 | 225,668,901 | 530 | BC039356  INTRON.3 | 7 | 36 | 50 | I/N | 0.11 |
| 3 | 125,889,195 | 319 | KALRN  INTRON.18 | 5 | 9 | 79 | I/N | 0.14 |
| 3 | 171,433,718 | 328 | PRKCI  INTRON.1 | 9 | 25 | 59 | I/N | 0.54 |
| 10 | 128,951,622 | 294 | DOCK1  INTRON.29 | 50 | 17 | 23 | I/N | 0.04 |
| 17 | 63,939,503 | 181 | WIPI1  INTRON.8 | 5 | 81 | 4 | I/N | 1.00 |
| 13 | 92,904,846 | 320 | GPC6  INTRON.1 | 9 | 27 | 56 | I/N | 0.30 |
| 8 | 27,351,899 | 542 | PTK2B  INTRON.18 | 43 | 40 | 8 | I/N | 0.15 |
